# Preclinical development of a replication-competent vesicular stomatitis virus-based Lassa virus vaccine candidate advanced into human clinical trials

**DOI:** 10.1016/j.ebiom.2025.105647

**Published:** 2025-03-28

**Authors:** Christopher L. Cooper, Gavin Morrow, Maoli Yuan, Thomas S. Postler, Maxwell L. Neal, Robert W. Cross, Courtney Woolsey, Krystle N. Agans, Viktoriya Borisevich, Ryan P. McNamara, Caroline Atyeo, Vicky Roy, Daritza Germosen, Fuxiang Hou, Shui L. Li, Lucia Reiserova, Yesle Choi, Aaron Wilson, Denise Wagner, Olivia Wallace-Selman, Alexei Carpov, Fuqiang Geng, Deborah J. Frederick, Joanne DeStefano, Anne M. Ercolini, Adrian S. Enriquez, Kathryn M. Hastie, Suzane Ramos da Silva, Eddy Sayeed, John W. Coleman, Andrew Kilianski, Galit Alter, Erica Ollmann Saphire, John D. Aitchison, Thomas W. Geisbert, Swati B. Gupta, Mark B. Feinberg, Christopher L. Parks

**Affiliations:** a IAVI, Vaccine Design and Development Laboratory, Jersey City, NJ 07302, USA; b IAVI, New York, NY 10004, USA; c Galveston National Laboratory, University of Texas Medical Branch, Galveston, TX 77555, USA; d Department of Microbiology and Immunology, University of Texas Medical Branch, Galveston, TX 77555, USA; e Ragon Institute of Massachusetts General Hospital, Massachusetts Institute of Technology and Harvard, Cambridge, MA 02139, USA; f Center for Global Infectious Disease Research, Seattle Children's Research Institute, Seattle, WA 98109, USA; g Departments of Pediatrics and Biochemistry, University of Washington, Seattle, WA 98109, USA; h Center for Vaccine Innovation, La Jolla Institute for Immunology, La Jolla, CA 92037, USA

**Keywords:** Lassa virus, Vesicular stomatitis virus vector, Clinical vaccine candidate, Neutralizing antibodies

## Abstract

**Background:**

Lassa fever (LF) is a zoonotic haemorrhagic disease caused by Lassa virus (LASV), which is endemic in West African countries. The multimammate rat is the main animal reservoir and its geographic range is expected to expand due to influences like climate change and land usage, and this will place larger parts of Africa at risk. We conducted preclinical development on a promising experimental vaccine that allowed its advancement into human trials.

**Methods:**

The LF vaccine is based on a vesicular stomatitis virus (VSV) vector in which the VSV glycoprotein (G) was replaced with the LASV glycoprotein complex (GPC). Earlier studies showed that this vaccine (VSVΔG-LASV-GPC) was efficacious in macaques, thus we regenerated VSVΔG-LASV-GPC using laboratory and documentation practices required to support vaccine manufacturing and human trials. The efficacy of the clinical vaccine candidate was assessed in cynomolgus macaques and more extensive immunologic analysis was performed than previously to investigate immune responses associated with protection.

**Findings:**

A single VSVΔG-LASV-GPC vaccination elicited innate, humoural and cellular immune responses, prevented development of substantial LASV viraemia, and protected animals from disease. Vaccinated macaques developed polyfunctional antibodies and serum was shown to neutralize virus expressing GPCs representative of geographically diverse LASV lineages.

**Interpretation:**

The VSVΔG-LASV-GPC clinical candidate elicited immunity that protected 10 of 10 vaccinated macaques from disease supporting its use in a clinical development program, which recently progressed to phase 2 clinical trials. Moreover, immunologic analysis showed that virus-neutralizing serum antibodies likely played a role in preventing LASV disease in vaccinated macaques.

**Funding:**

This work was supported by the 10.13039/100016302Coalition for Epidemic Preparedness Innovations (CEPI), The National Institute of Allergy and Infectious Diseases (NIAID)/10.13039/100000002National Institutes of Health (NIH), The Bill and Melinda Gates Global Vaccine Accelerator Program, the 10.13039/100000861Burroughs Wellcome Fund, and financial gifts and support by Nancy Zimmerman, Mark and Lisa Schwartz, and Terry and Susan Ragon.

**Research in context**

**Evidence before this study**

Multiple vaccine candidates for prevention of LF have been developed including those based on DNA, mRNA, virus-like particles, nanoparticles, attenuated viruses, and viral vectors, but few phase 1 clinical trials have been completed to date and no prophylactic vaccine is licensed for use. When the experimental Ebola virus vaccine (VSVΔG-ZEBOV-GP) was originally developed that later became ERVEBO®, a similar prototype LF vaccine was generated with the same VSV-based vector technology. Like VSVΔG-ZEBOV-GP, a single vaccination with the LASV vaccine was shown to be consistently efficacious in cynomolgus macaques exposed to high doses of LASV, which provided a strong rationale for advancing the vaccine candidate for evaluation in human trials. In this report we describe preclinical development that has allowed the LASV vaccine candidate (VSVΔG-LASV-GPC) to advance into phase 1 and 2 clinical trials.

**Added value of this study**

This report describes generation of a VSVΔG-LASV-GPC vaccine virus seed that supported advancement of the vaccine candidate for manufacturing and phase 1 and 2 clinical trials. The efficacy of the clinical vaccine candidate was assessed in cynomolgus macaques and more extensive immunologic analysis was performed than done previously to investigate immune responses associated with protection from LASV. Like described for the original research vaccine, a single dose of 2 × 10^7^ plaque-forming units (PFU) of VSVΔG-LASV-GPC protected vaccinated macaques, and as shown here a lower dose (2 × 10^5^ PFU) was equally effective, which provided guidance for the dose range to be used in humans. Furthermore, virologic, immunologic, and blood transcriptome analyses showed that either dose of VSVΔG-LASV-GPC elicited innate, humoural and cellular immune responses and prevented development of substantial LASV viraemia. The immunologic and transcriptome analyses provided some informative new data about the nature of the immune responses elicited by the VSV-based LASV vaccine, including 1) vaccination rapidly stimulated expression of genes associated with the interferon response that also have been shown to be modulated by ERVEBO vaccination; 2) vaccination elicited anti-GPC serum IgG that could direct multiple effector functions *in vitro* such as cellular phagocytosis, natural killer (NK)-cell activation, and complement deposition; and importantly 3) vaccinated macaques developed serum antibodies that could neutralize virus expressing GPCs representative of geographically diverse LASV lineages.

**Implications of all the available evidence**

Patients that recover from LASV infection rapidly mount a strong cellular immune response that is associated with their recovery while detectable virus-neutralizing antibodies (nAbs) develop late in convalescence indicting that cellular immunity is correlated with viral clearance. In contrast, a notable feature of the response to VSVΔG-LASV-GPC vaccination is induction of nAbs suggesting that they may play a central role in disease prevention by vaccination. Furthermore, our data indicate that VSVΔG-LASV-GPC elicits serum antibodies that can neutralize virus expressing GPCs representative of diverse LASV lineages implying that vaccination will have the potential to prevent disease due to LASV strains circulating in different geographic regions. In a companion manuscript, mapping of regions bound by serum antibodies by electron microscopy indicates that conserved GPC structures are targeted by serum nAbs providing a molecular basis for the broad neutralization activity. Thus, serum nAb activity will be one of the key markers to follow in the phase 1 and 2 clinical trials conducted with VSVΔG-LASV-GPC.

## Introduction

Lassa fever (LF) is a zoonotic haemorrhagic disease caused by Lassa virus (LASV; species *Mammarenavirus lassaense*), an enveloped RNA virus in the *Arenaviridae* family.^1^ LASV is endemic in multiple countries in West Africa, where its primary reservoir is the multimammate rat (*Mastomys natalensis*).^1^ The geographic range of the animal hosts places large populations at risk of LASV infection, with some estimates of up to four million infections per year^2^ and this is projected to increase substantially due to the influence of land use, climate change, and population growth.^3^ Additionally, there is the threat of LASV being used for bioterrorism.^1^

LASV infection in people causes a range of clinical outcomes.^4^ Many LASV infections are asymptomatic, and others present symptoms like other common infections, such as fever, malaise, myalgia, and nausea.^4^ Acute haemorrhagic fever occurs in about 20% of cases, and mortality is high for hospitalized patients, as well as pregnant women and in particular their foetuses.^1^^,^^4^ Furthermore, LASV infection can cause permanent hearing loss regardless of LF severity.^1^^,^^4^ Because of its potential to cause a public health emergency, LASV is included in the World Health Organization's (WHO’s) R&D Blueprint for development of diagnostics, vaccines, and treatments.^5^

Immune responses that contribute to recovery from LF are not completely understood. In people who recover from LF, there is strong activation of antigen-specific peripheral blood T cells indicating that cellular immunity plays an important role in natural acquired control and clearance of LASV infection.^6^^,^^7^ In contrast, neutralizing serum antibodies (nAbs) have been reported to develop slowly in infected patients and may not increase substantially until late convalescence.^6^^,^^7^ The delayed induction of nAbs may be due to acute LASV infection causing dysregulation of the humoural immune response and further suggests that antibody-mediated neutralization is not a main contributor to viral clearance in unvaccinated individuals.^6^^,^^7^ Consistent with observations in infected people, recovery of macaques from experimental LASV infection has been shown to correlate with development of rapid and potent innate and cellular immunity, provided it did not lead to an excessive inflammatory response.^8^^,^^9^

Although nAbs may not develop rapidly enough to play a central role in clearance of a natural LASV infection in an immunologically naïve person, it is important to note that monoclonal antibodies (mAbs) specific for the envelope glycoprotein complex (GPC) that potently neutralize LASV infectivity have been isolated from recovered patients. Combinations of these anti-GPC nAbs administered as a post-exposure therapeutic have been able to rescue macaques when administered as much as 8 days after LASV infection was initiated by intramuscular (IM) injection or virus application to the intranasal mucosa.^10^, ^11^, ^12^ The preclinical efficacy of these nAbs suggests that a prophylactic vaccine capable of inducing similar antibodies should protect from LASV disease.

Currently, a prophylactic LF vaccine is not authorized for use in people. Preclinical and clinical research is being conducted on multiple types of LASV vaccine candidates including those based on nucleic acids, virus-like particles (VLPs), nanoparticles, virus replicons, live-attenuated viruses, and various types of viral vectors.^1^^,^^13^, ^14^, ^15^ Importantly, experimental vaccines based on chemically inactivated LASV particles were shown to elicit immune responses in macaques but failed to protect the animals from disease following subsequent exposure to LASV,^16^ indicating that the characteristics of the delivered viral immunogens play a prominent role in the development of protective immunity. In fact, structural studies with the potent nAbs like those mentioned above indicate that they bind structural epitopes unique to the native multimeric LASV envelope GPC,^17^, ^18^, ^19^ which is the only immune system target exposed on the virion surface.^20^ Thus, vaccines that can deliver immunogenic native-like GPC structures are important to develop and test.

Historically, effective vaccines based on live-attenuated enveloped viruses like measles, mumps, and yellow fever have reliably induced serum nAbs correlated with protection because they expose the immune system to native viral envelope glycoproteins in the context of a mild but immunostimulatory viral infection.^21^ Although the live attenuated virus approach can be very effective, it would be challenging to develop a safe and efficacious live vaccine from highly virulent LASV. The Zaire ebolavirus (ZEBOV; now known as Ebola virus or EBOV^22^) vaccine that is licensed for use in people and is marketed by Merck Sharp & Dohme as ERVEBO® provides a valuable alternative live viral vaccine strategy.^23^^,^^24^ ERVEBO is based on a recombinant vesicular stomatitis virus (VSV) in which the gene encoding the single VSV envelope glycoprotein (VSV G) was replaced with a gene that codes for the native EBOV glycoprotein (GP).^23^^,^^25^ The resulting replication-competent chimeric virus, called VSVΔG-ZEBOV-GP, relies on EBOV GP to functionally substitute for VSV G and mediate host cell attachment and entry. Although substitution of VSV G with the heterologous EBOV GP changes cellular receptor usage and attenuates VSV replication,^25^ multiple preclinical studies have shown that a single IM injection with live VSVΔG-ZEBOV-GP safely elicits protective immunity in macaques.^23^^,^^26^^,^^27^ Furthermore, clinical trials have shown that VSVΔG-ZEBOV-GP was very effective when used during the large EBOV outbreak that began in 2014,^24^ and that a single vaccination was shown to elicit both innate and adaptive immune responses^28^ associated with rapid development of protective immunity in people.^29^ Overall, the cumulative preclinical and clinical experience with VSVΔG-ZEBOV-GP indicates that the live VSVΔG chimeric virus design is effective in eliciting antibodies against the complex EBOV GP that are correlated with efficacy,^27^^,^^30^ and that the VSVΔG-based technology should be more broadly applicable to vaccines against other viral glycoproteins including LASV GPC. In fact, preclinical research has shown that an experimental VSVΔG-based LASV vaccine (VSVΔG-LASV-GPC) is consistently efficacious in macaques, providing a strong rationale to advance development of a prophylactic VSVΔG-based LASV vaccine for use in humans.^31^, ^32^, ^33^, ^34^

Below we describe preclinical development of the VSVΔG-LASV-GPC vaccine for use in people. Evaluation of this candidate in a multicentre phase 1 clinical trial is complete (ClinicalTrials.gov: NCT04794218 and^15^) and a phase 2 trial has begun (ClinicalTrials.gov: NCT05868733 and^15^). The preclinical data described below demonstrate that this vaccine is efficacious in cynomolgus macaques after a single IM injection with 2 × 10^7^ plaque-forming units (PFU) or a 100-fold lower dose, a dose range realistic for use in people.^24^ Notably, vaccinated macaques developed LASV GPC-binding antibodies with multi-functional characteristics, and importantly, immune serum was found to neutralize homologous and heterologous VSVΔG-LASV-GPC chimeric viruses. Vaccination elicited a strong type-I interferon (IFN-I) response shortly after vaccination, similar to what has been reported for VSVΔG-ZEBOV-GP,^28^^,^^35^^,^^36^ suggesting the possibility that IFN-I signature may serve as a biomarker for successful vaccination with VSVΔG-based vaccines more broadly.

## Methods

### Ethics

Vaccination was performed in ABSL-2 laboratories and LASV challenge was conducted in ABSL-4 laboratories at the University of Texas Medical Branch (UTMB) at Galveston. This research was conducted in accordance with the ethical guidelines of the *Guide for the Care and Use of Laboratory Animals: Eighth Edition*. The study design was reviewed and approved by the UTMB Institutional Care and Use Committee (IACUC) under protocol and the Institutional Biosafety Committee (IBC) under notification of use number 2020063. The study was performed in a facility accredited by the Association for Assessment and Accreditation of Laboratory Animal Care International (AAALACi) (Accreditation #000870, July 2023). The program has an approved animal welfare assurance on file with NIH, Office of Laboratory Animal Welfare (#D16-00202 [formerly A3314-01]) and is registered with the USDA/APHIS/Animal care (#R-74-0073).

### Cell culture

Vero cells used in all phases of this work were from a cell bank that was established and qualified for use in human vaccine production. The qualified cell bank was generated using cells from the WHO working cell bank (WHO 10–87) deposited at the European Collection of Authenticated Cell Cultures (Vero[WHO], ECACC 88020401). The qualified cell bank has been used to develop other VSV-based clinical vaccine candidates.^37^^,^^38^ Cell monolayers were cultured in Dulbecco’s modified Eagle medium (DMEM) supplemented with 4 mM L-glutamine and 10% of gamma-irradiated foetal bovine serum (FBS) (all MilliporeSigma). Cells were incubated at 37 °C with 5% CO_2_ and 85% humidity. Monolayers were dissociated for subculturing with TrypLE™ Select (Gibco, ThermoFisher Scientific).

### Recombinant VSV

The VSVΔG-LASV-GPC genomic clone, encoding GPC from LASV lineage IV (Josiah), was kindly provided by the Public Health Agency of Canada (PHAC).^25^ Recovery of VSVΔG-LASV-GPC from plasmid DNA was performed by electroporation of Vero cells, as previously described using the genomic clone and 6 supporting plasmids controlled by the hCMV promoter that expressed the bacteriophage T7 RNA polymerase gene and VSV genes optimized using a human codon bias that encode VSV nucleocapsid protein (N), phosphoprotein (P), matrix protein (M), glycoprotein (G) and large protein (L).^37^^,^^38^ Three days post-electroporation, medium supernatant was harvested and used in subsequent infections. Two days later, medium was collected and stored in aliquots at ≤ −60 °C. After samples of the virus were subjected to RNA extraction and nucleotide sequencing to confirm the identity of the recovered virus population, three rounds of plaque isolation were performed.^37^^,^^38^ Virus from multiple individual plaques were amplified in Vero cells monolayers, followed by RNA extraction and sequencing analysis.^38^ Lead candidates were then subjected to two additional rounds of plaque isolation before they were used to infect Vero cells cultured in 5-layer cell stacks (Corning). At 40–48 h after infection, medium containing virus was harvested and clarified at 400×*g*. The clarified medium was aliquoted and stored at ≤ −60 °C. Further evaluation of the candidates is described in the following section. The selected candidate for clinical development was then designated as the pre-master virus seed (preMVS) that was later used to support manufacturing of clinical trial material (ClinicalTrials.gov: NCT04794218).

VSVΔG-LASV-GPC recombinants also were developed for use in a plaque reduction neutralization test (PRNT) described in the corresponding section below. GPC-coding sequences representing LASV lineages I–V and VII from different West Africa geographic regions (Genbank IDs in Supplementary Table S2) were optimized using a codon frequency consistent with VSV and use of synonymous codons to disrupt any nucleotide sequence that might interfere with VSV transcription or replication.^39^ The optimized GPC genes were synthesized by GenScript and inserted between the VSV M and L genes of a VSV Indiana genomic clone described before.^39^ Recovery, propagation, and characterization of recombinant viruses were performed similarly as described above. A VSVΔG chimera expressing the MARV Musoke GP was prepared analogously and was utilized as a vector control for assays described below.^38^

### Production of purified VSV recombinants

Vaccine material used in the preclinical efficacy study was produced by infecting Vero cell monolayers with the preMVS as described previously.^38^ After harvesting medium from infected cells, virus was concentrated and purified by tangential flow filtration (TFF; Repligen). Briefly, Vero cells were seeded in a 5-layer Cell Stack (Corning) with high-glucose DMEM (ThermoFisher) supplemented with 2 mM L-glutamine and 10% FBS (both MilliporeSigma), after which they were incubated for 48–72 h until the monolayer was nearly confluent. The cell monolayer was washed three times with DMEM lacking FBS before adding 375 ml of Virus-Production Serum-Free Medium (VP-SFM; ThermoFisher Scientific), supplemented with 4 mM L-glutamine and containing VSVΔG-LASV-GPC preMVS to achieve a multiplicity of infection (MOI) of 0.001. At 44 h after infection, culture medium containing virus was harvested and subsequently clarified by sequential filtration with a 1.2-μm filter (Sartorius Sartopure) followed by a 0.8/0.45-μm depth filter (Pall Corporation). TFF was used to concentrate and further purify the virus with a 750-kD hollow-fibre membrane (GE Healthcare) at 70 ml/min. Benzonase treatment (200 U/ml; MilliporeSigma) in the presence of 1.5 mM of MgCl_2_ was performed for 30 min at room temperature to digest contaminating nucleic acid while circulating through the membrane. Diafiltration then was performed with Hank’s balanced salt solution (HBSS) containing CaCl_2_, MgCl_2,_ and MgSO_4_ (pH 7.2; ThermoFisher Scientific) that was supplemented with 15% trehalose (MilliporeSigma). Transmembrane pressure was kept lower than 2.0 psi during the whole process. The final solution was filtered with a 0.2-μm Steripak-GP 10 filter unit (MilliporeSigma), aliquoted, and stored at ≤ −60 °C.

VSVΔG-LASV-GPC (Lineages I–V, VII) and VSVΔG-MARV-GP (Marburg virus GP) used in the PRNT were purified by ultracentrifugation using a sucrose cushion to remove cellular and viral protein contaminants released by the cytolytic VSV infection. Medium harvested from infected Vero cell monolayers was clarified by centrifugation (30 min, 4 °C, 900×*g*) then 27 ml was layered on top of 10 ml sucrose cushions (20% sucrose prepared in phosphate-buffered saline [PBS]) in ultracentrifuge tubes (Beckman–Coulter swinging bucket SW28). Virus was collected by centrifugation at 18,000 rpm (58,000×*g*) for 2 h at 4 °C after which the liquid was removed completely. Virus was resuspended in formulation buffer composed of HBSS containing CaCl_2_, MgCl_2,_ and MgSO_4_ pH 7.2 from ThermoFisher Scientific that was supplemented with 15% trehalose (MilliporeSigma). Purified virus was analysed with quality control assays described below.

### Plaque assays for VSVΔG-LASV-GPC potency

Infectious VSVΔG-LASV-GPC was quantified by plaque assay. Plaque assays were performed using Vero cell monolayers that were cultured in six-well culture plates (Falcon) containing DMEM supplemented with 10% FBS as described above. Ten-fold dilutions of the viral stock were prepared in DMEM containing 2% FBS and 0.5 ml of each dilution were used to infect cell monolayers. After infection proceeded for 1 h, the virus inoculum was removed, and cells were overlaid with Minimal Essential Medium (MEM; Gibco ThermoFisher Scientific) containing 0.8% agarose (SeaPlaque™ agarose, Lonza) and 2% FBS. Plaques were visualized 48 h post-infection by fixing the cell monolayers with 7% formaldehyde and then staining with 0.3% crystal violet prepared in Milli-Q® ultrapure water (both from MilliporeSigma).

### VSVΔG-LASV-GPC genome copy quantification in vaccine material

RNA was extracted from 100 μl of purified virus following manufacturer instructions (RNeasy Mini Kit, Qiagen). VSV genome copies were quantified by real-time quantitative PCR (RT-qPCR) specific for the VSV N gene.^39^ Briefly, duplicate reverse transcriptase reactions were performed using 15 μl of purified RNA per reaction; 10 μl of a cocktail composed of reagents from the Sensiscript Reverse Transcriptase kit (Qiagen) including 1× reverse transcription buffer, 0.5 mM of each dNTP, 10 U RNase Inhibitor (Invitrogen, Thermofisher Scientific), and 10 U Sensiscript reverse transcriptase; and a VSV N-specific forward primer (400 nM, 5′-CGGAGGATTGACGACTAATGC-3′ [Integrated DNA Technologies]) that anneals to the negative-sense genomic RNA. Reverse transcription was performed at 50 °C for 45 min and heat-inactivated at 95 °C for 2 min. Twenty-five μl of the reaction was used for the qPCR with the addition of 25 μl of a reagent mix composed of 1× QuantiTect Multiplex PCR Master Mix (Qiagen), 400 nM each of VSV N-specific reverse primer (5′-ACCATCCGAGCCATTCGA-3′) and VSV N-specific forward primer, and 200 nM 6-carboxyfluorescein (FAM)-labelled minor groove binder (MGB) probe (5′-6FAM-CGCCACAAGGCAG-MGB-3′; ThermoFisher Scientific). An AriaMx Real-Time PCR Instrument (Agilent) was used for amplification and detection with the following conditions: 15 min at 95 °C followed by 45 cycles of 15 s at 94 °C and 60 s at 60 °C. Results from duplicate test samples were averaged and genome copy numbers were interpolated from a curve generated with known RNA standards.^39^

### Sanger sequencing

VSVΔG-LASV-GPC whole-genome sequencing was performed by generating cDNA from purified RNA (RNeasy Mini Kit, Qiagen) using the SuperScript III one-step RT-PCR System (ThermoFisher Scientific), with seven overlapping cDNA fragments covering the complete VSVΔG-LASV-GPC genome.^38^ RT-PCR was performed at 55 °C for 30 min, followed by 94 °C for 2 min and 40 cycles of 94 °C for 15 s, 57.5 °C for 30 s, and 68 °C for 3 min with final extension at 68 °C for 5 min. Sanger sequencing was performed with BigDye Terminator v3.1 Cycle Sequencing Kit and BigDye XTerminator™ Purification Kit with an ABI 3500XL Genetic Analyser (all ThermoFisher Scientific).

### Additional vaccine quality controls

Vaccine material was subjected to the following analyses: a qPCR assay for *Mycoplasma* spp. using MycoSEQ™ Mycoplasma Detection Kits (Applied Biosystems); quantification of cell-host DNA with resDNASEQ™ Quantitative Vero DNA Kits (Applied Biosystems); and total protein content by Bradford protein assay. Sterility testing was conducted with Luria–Bertani (LB) agar, malt extract agar, blood agar, and chocolate agar plates (Teknova) incubated at 37 °C for 7 days.

### Flow virometry

Viral particles in the purified vaccine material were quantified with an A60-MicroPLUS flow virometer (Apogee Flow Systems Ltd) using purified Milli-Q water as sheath fluid. Briefly, the sample was diluted 1:300 in sterile HBSS and run at 1.5 μl/min with the autocycler set to 200,000 total events. A 405-nm violet laser was set to 150 mW and a Large-Angle Light Scatter detector was used to resolve the peak profile.

### Flow cytometry

Flow cytometry was used to assess cell-surface expression of GPC and intracellular expression of VSV N.^38^ Adherent infected cells were detached from plates at 40–48 h after infection with VSVΔG-LASV-GPC by scraping them into a wash solution containing PBS (ThermoFisher Scientific) supplemented with 0.5% bovine serum albumin (BSA; MilliporeSigma) (PBS/BSA). The cell suspensions were then distributed into a 96-deep-well tissue culture plate (Nunc) before collection by low-speed centrifugation for 5 min at 860×*g*. For staining of GPC on the cell surface, the cell pellets were resuspended in PBS/BSA containing human mAbs specific for LASV GPC^40^ (all from Zalgen Labs: GP2 LASV HuMAb 8.4F [SKU Ab00084F], GP2 LASV HuMAb 24.6C [SKU Ab00246C], and GPC LASV HuMAb 25.10C [SKU Ab02510C])^40^ at a final concentration of 1 μg/ml before incubation at room temperature for 25 min. The anti-HIV-1 Env antibody PGT-145^41^ used as a control was generated in-house at IAVI. Next, the cells were collected by centrifugation, resuspended in PBS/BSA, and centrifugation was repeated. The pelleted cells then were resuspended in Cytofix/Cytoperm Solution (BD Biosciences) and incubated for 20 min at 4 °C in the dark per the manufacturer’s recommendations to prepare cells for intracellular staining. The permeabilized cells were collected by centrifugation and resuspended in Perm/Wash Buffer (BD Biosciences) before repeating centrifugation. To stain intracellular VSV N, the cells were resuspended in Perm/Wash Buffer containing anti-VSV N mouse mAb (10G4, Kerafast cat.-# EB0009^42^) at a final concentration of 1 μg/ml and were then incubated in the dark at room temperature for 25 min. Following incubation, the cells were collected and washed with Perm/Wash Solution as described above, after which the cells were resuspended with Perm/Wash Solution containing labelled secondary antibodies including goat anti-human Alexa 555 and goat anti-mouse Alexa 647 (ThermoFisher Scientific). Cells were incubated in the dark at room temperature for 25 min before the free antibodies were removed by centrifugation and washing with Perm/Wash Buffer. Cells were resuspended in Perm/Wash Buffer and flow cytometry was performed with a BD SORP LSRII flow cytometer (BD Biosciences). Results were analysed with FlowJo software (BD Biosciences/Treestar, version 10.6.2).

### Analysis of virus particles in purified vaccine material by electron microscopy

Three μl of purified VSVΔG-LASV-GPC was adsorbed onto a continuous carbon film on 400 mesh copper grids (Electron Microscopy Services) that were glow discharged for 15 s. Excess solution was manually flicked off the grid and then washed three times with 50 mM HEPES (MilliporeSigma) with 50 mM NaCl (ThermoFisher Scientific). Next, adsorbed VSVΔG-LASV-GPC was fixed with 2.3% glutaraldehyde (Electron Microscopy Services) for 20 min. Excess solution was manually flicked off and subsequent staining of the grid was performed using 2% methylamine tungstate pH 6.8 (VitroEase, ThermoFisher Scientific). Excess stain solution was flicked off and the grid was air dried before imaging on a FEI Titan Halo electron microscope with a K3 direct electron detector (Gatan) at the magnification of 58,000× for a pixel size of 1.7 Å/pixel. All steps in the negative stain grid preparation were done with the tweezers holding the grid resting on ice. All 2D and 3D image processing was performed using cryoSPARC v.4.0.^43^ Contrast Transfer Function (CTF) correction of negative stain electron micrographs was done using Patch CTF in cryoSPARC. Particle projections were manually picked and then sorted into reference-free 2D class averages. Selected particle projections were then used to generate ab-initio reconstructions that underwent several rounds of 3D refinement, first without symmetry and then using C3 symmetry. Visualization and colouring of negative stain 3D reconstructions was performed using ChimeraX.^44^

### Western blot

GPC expression was evaluated by Western blot as described before.^37^ Viral proteins from purified denatured VSVΔG-based vaccines were resolved by 4–12% reducing LDS-PAGE, transferred to nitrocellulose membrane, and probed with human mAb specific for GP1 (Zalgen GP1 LASV HuMAb 3.3B[SKU Ab00033B]) or for GP2 (GP2 LASV HuMAb 22.5D [SKU Ab00225D]).^40^ VSV N was detected using a rabbit polyclonal serum produced at IAVI.^39^ Secondary antibodies linked to horseradish peroxidase (Jackson ImmunoResearch) and the Enhanced Chemiluminescence System (Amersham Biosciences) were used to produce a signal detected with a ChemiDoc imager (Bio-Rad). Protein ladders were a 1:1 mixture of MagicMark™ XP Western Protein Standard (ThermoFisher Scientific) and Novex™ Sharp Pre-stained Protein Standard (ThermoFisher Scientific).

### VSVΔG-LASV-GPC vaccination

Thirteen healthy treatment-naïve adult captive-bred cynomolgus macaques (*Macaca fascicularis*) of Chinese origin 4–6 years of age and weighing 3.2–4.6 kg were randomly assigned to two vaccine groups of five animals each and a control group of three animals. Nonhuman primate (NHP) test groups were attempted to be sex balanced with assignment of three females and two males per vaccine cohort. The control group consisted of one female and two males. Animals were then randomly assigned to each cohort in Microsoft Excel using the RAND formula and singly housed. Test groups (n = 5) were administered either a single dose of 2 × 10^7^ PFU VSVΔG-LASV-GPC or a lower dose of 2 × 10^5^ PFU, both by IM injection. Animals were vaccinated once with 1 ml of virus injected into the caudal thigh. The control group (n = 3) received an equivalent volume of vaccine diluent (5% trehalose in PBS) delivered IM. Titres from diluted unused vaccine virus were determined by plaque assay to confirm the expected formulation dose for each vaccination.

Blood was drawn by femoral venipuncture and collected into sodium heparin vacutainer tubes (BD Biosciences) prior to and on days 1, 3, 10 and 27 after vaccination. Blood tubes were centrifuged at 800×*g* for 10 min to remove plasma before peripheral blood mononuclear cell (PBMC) isolation by density gradient centrifugation on Ficoll-Hypaque (GE Healthcare) using Accuspin tubes (MilliporeSigma) according to the manufacturer’s instructions. The tubes were centrifuged at 800×*g* and room temperature for 15 min, and the resulting buffy coat was collected. Cells were washed once in Roswell Park Memorial Institute 1640 (RPMI) medium supplemented with 10% FBS, 100 U/ml penicillin, 100 μg/ml streptomycin, and 1% L-glutamine (R10; all ThermoFisher Scientific) and treated briefly with ammonium chloride potassium red cell lysis buffer (ACK; ThermoFisher Scientific) to remove erythrocytes. PBMCs were then centrifuged at 250×*g* for 10 min, washed twice with R10 medium, and enumerated with a TC20 Automated Cell Counter (Bio-Rad). PBMCs were stored in FBS containing 10% dimethyl sulfoxide (DMSO; MilliporeSigma) in a liquid-nitrogen freezer. Serum was isolated from blood collected in serum separator tubes (SSTs; BD Biosciences) by centrifugation at 800×*g* for 10 min. Serum and plasma aliquots were stored at −20 °C until used.

### Detection of VSVΔG-LASV-GPC RNA

VSVΔG-LASV-GPC RNAemia was assessed by RT-qPCR based on VSV N as described above. RNA was purified from plasma derived from blood collected on days 1, 3 and 10 post-vaccinations. Briefly, virus from 1.0 ml of plasma was collected by centrifugation at 61,973×*g* (Allegra 64R; Beckman Coulter) for 60 min at 4 °C. The virus pellet was then processed using the RNeasy Mini Kit (Qiagen) by suspending virus in a solution containing 300 μl of lysis buffer from the kit supplemented with 142 mM of 2-mercaptoethanol (Bio-Rad Laboratories), and 1 mg/ml of proteinase K (ThermoFisher Scientific). Samples were digested at 56 °C for 1 h, after which RNA was purified using spin columns and the QIAvac 24 Plus vacuum system (Qiagen) following the RNeasy Mini Kit protocol. RNA was eluted in 50 μl of RNase-free water supplemented with 1 mM dithiothreitol (MilliporeSigma) and 1 U/ml RNAseOUT (Thermofisher Scientific). Positive samples were defined as ≥200 genome copies/ml of plasma.

### GPC-binding enzyme-linked immunosorbent assay (ELISA)

Anti-GPC serum IgG binding titres were quantified by ELISA using a soluble GP1–GP2 fusion protein (linked LASV-GP, lineage IV; Zalgen Labs) expressed from Drosophila S2 cells.^17^^,^^40^ Corning 96 well half-area microplates were coated with 1 μg/ml protein in ELISA Coating Buffer (Biolegend) overnight at 4 °C after which the plates were washed three times with ELISA wash buffer (PBST; PBS [Corning] with 1% Tween-20 [VWR]) before blocking with PBS containing 3% BSA for 2 h at 37 °C. After blocking, the plates were washed with ELISA wash buffer and were then incubated with serial dilutions of macaque sera prepared in dilution buffer (PBS plus 1% BSA) for 1 h at 37 °C. Following incubation, plates were washed with PBST and were then incubated for 1 h at 37 °C with 1:4000 dilution of horseradish peroxidase (HRP)-conjugated anti-monkey IgG secondary antibody (Jackson ImmunoResearch) prepared in dilution buffer. After removing the secondary antibody and washing the plates with ELISA wash buffer, signal was developed using 1-Step Ultra Tetramethylbenzidine (TMB) Substrate (Thermofisher Scientific) for 10 min at room temperature before the reaction was stopped with 2N sulfuric acid (VWR). Absorbance at 450 nm was read using a SpectraMax Plus Microplate Reader (Molecular Devices). Binding antibody endpoint titres were calculated using GraphPad Prism 8 software using 4-parameter non-linear curve fitting.

### Enzyme-linked immunospot (ELISpot)

LASV GPC-specific T cells in peripheral blood were quantified by IFN-γ ELISpot assay^45^ using the MabTech Monkey IFN-gamma ELISPOT Basic kit (Mabtech). Ninety-six-well multiscreen ELISpot plates (MilliporeSigma) were coated overnight at 4 °C with anti-IFN-γ antibody. The following day, plates were washed with PBS containing 0.05% Tween-20 (ThermoFisher Scientific) and were then incubated in R10 medium for 1 h at 37 °C, then rinsed with R10 medium before addition of 200,000 PBMCs in R10 medium and recombinant LASV GPC protein (Zalgen Labs), LASV GPC, or VSV N peptide pools (Genscript) at a concentration of 2.5 μg per peptide per ml. All peptide pools consisted of 15-mers overlapping by 11 amino acids. PBMCs incubated with DMSO or Staphylococcal enterotoxin B (SEB; MilliporeSigma) served as negative and positive controls, respectively. Plates were incubated for 18–20 h at 37 °C in 5% CO_2_, after which they were washed and developed according to the manufacturer’s instructions using an HRP-labelled secondary antibody and TMB substrate. IFN-γ spots were counted using an automated ELISpot reader (Cellular Technology Limited). Antigen-specific responses were determined by subtracting the number of spots in DMSO-treated from peptide-treated wells. Results are shown for average spots per 10^6^ PBMCs from duplicate wells.

### PRNT

Virus-neutralizing anti-GPC serum antibodies (nAbs) were quantified using VSVΔG-LASV-GPC recombinants expressing GPCs from different LASV lineages (Supplementary Table S2) and VSVΔG-MARV-GP as a negative control. Viruses used for PRNT were purified using centrifugation through a sucrose cushion as described above. Serum collected 27 days after vaccination was heat-inactivated at 56 °C for 30 min, after which it was clarified by centrifugation at 9300×*g* for 10 min. Clarified serum diluted serially from 1:20 to 1:2560 was incubated for 1 h at 37 °C with VSVΔG-LASV-GPC before the serum-virus mixture was used to infect Vero cell monolayers in 96-well plates. The amount of VSVΔG-LASV-GPC used was titrated to yield approximately 100 plaques per well under mock conditions. Following incubation for 2 h at 37 °C, cells were overlaid with DMEM containing 1% FBS and 0.5% methylcellulose (both ThermoFisher Scientific). Plaques were allowed to develop for 24 h at 37 °C before the methylcellulose overlay was removed and the monolayer was washed once with PBS in preparation for fixation with ice-cold methanol for 1 h at room temperature. After removing the methanol, plates were air-dried before being rehydrated for 10 min with PBS containing 1% BSA and 1% goat serum (GS; MilliporeSigma) followed by blocking with 5% BSA/5% GS in PBS for 1 h at room temperature. The blocking buffer was removed and then VSV N rabbit polyclonal antiserum^39^ diluted in 1% BSA/1% GS in PBS was applied to each well and incubated for 1 h at 37 °C. Plates were then washed three times with 1% BSA/1% GS in PBS before incubating for 1 h with goat anti-rabbit antibody labelled with Alexa 488 (ThermoFisher Scientific) in PBS with 1% BSA/1% GS. Wells were then washed three times with 1% BSA/1% GS in PBS and stained plaques were counted using a Gen5 imager (Agilent). The lowest serum dilution that decreased the number of plaques by at least 50% was reported.

### Systems serology

Systems serology was conducted as described earlier.^46^ To capture anti-GPC antibodies from serum, beads were linked to GP-link or GP1–GP2 complex stabilized in native-like prefusion form by a cysteine linkage,^17^^,^^40^ along with control antigens (influenza A virus haemagglutinin [HA] and Ebola virus GP, Sino Biological). Luminex beads were coupled with antigens by NHS-ester linkage through Sulfo-NHS and EDC (ThermoFisher Scientific) and incubated with serum dilutions in 384-well low-bind plates (Greiner-Bio) at 4 °C overnight with continuous shaking. The formed immune complexes were washed thrice on a 384-well HydroSpeed plate washer (Tecan) using Assay Buffer (1× PBS [Corning] pH 7.4, 0.1% w/v BSA [MilliporeSigma], 0.05% Tween-20). Beads were then incubated with phycoerythrin (PE)-conjugated detection antibodies directed against NHP antibody subclasses and isotypes (NHP Reagents Inc) diluted in Assay Buffer at room temperature for 1 h with continuous shaking. Beads were washed thrice with Assay Buffer, resuspended in 40 μl of QSOL buffer (Intellicyt, Sartorius) and then run on an Intellicyt iQue Screener Plus Flow Cytometer (Intellicyt, Sartorius). Gating was performed according to our previously validated SOP^47^ using iQue Forecyt v10.0.8341.

Binding of antigen-specific antibodies to custom-produced and purified Fc receptors (Duke Human Vaccine Institute) was done similarly to antibody profiling and according to our previously validated SOP.^48^ Purified Fc receptors were biotinylated using the BirA ligase (Avidity) and incubated with streptavidin-PE (Prozyme). Complexes were then added to Luminex beads that had been previously incubated with serum and washed. Binding was determined by flow cytometry.

For antibody-dependent cellular phagocytosis (ADCP), fluorescent neutravidin microspheres (ThermoFisher Scientific) were conjugated with target antigen and then blocked using 5% BSA in 1× PBS. Beads were washed with PBS and then incubated with diluted serum samples to allow for the formation of pre-immune complexes at 37 °C for 2 h. Beads were then washed before being added to 25,000 THP-1 cells (ATCC) per well in a 96-well plate (Corning) in RPMI medium supplemented with 10% FBS and β-mercaptoethanol (all MilliporeSigma). Plates were incubated at 37 °C overnight. Cells were then fixed, and phagocytosis was determined by gating for fluorescent-bead-positive cells on an Intellicyt iQue Screener Plus. Phagocytic score was defined as: (% bead positive cells) ∗ (gMFI of bead positive cells)/(10X gMFI of the first bead-positive peak).^47^

For antibody-dependent neutrophil phagocytosis (ADNP), antigen was coupled to fluorescent neutravidin microspheres similar to ADCP, and pre-immune complexes were allowed to form using serum samples as above. Primary leukocytes including neutrophils were obtained from blood freshly drawn into citrate dextrose tubes. After ACK (ThermoFisher Scientific) treatment, beads were added to leukocytes at a concentration of 50,000 cells/well in a 96-well plate and incubated at 37 °C for 1 h. Cells were then fixed and surface stained for CD66b, CD14, and CD3 (all BD Biosciences). Single fluorescent cells were quantified using an Intellicyt iQue Screen Plus and a phagocytic score was calculated.

Antibody-dependent complement deposition (ADCD) was quantified using antigen-coated beads incubated with serum for 2 h at 37 °C to allow for the formation of pre-immune complexes. Guinea pig complement (CedarLane) was diluted in veronal buffer (Boston Bioproducts) and incubated with the beads for 20 min at 37 °C. Beads were then washed with 5 mM EDTA in PBS and stained with anti-C3 FITC (MP Biomed). Complement deposition was quantified through fluorescence using an Intellicyt iQue Screener Plus.

Antibody-dependent natural killer cell activation (ADNKA) was performed on 96-well ELISA plates (ThermoFisher Scientific) containing immobilized target antigen(s). Serum samples were diluted in 5% BSA in PBS, added to the plates, and incubated at 37 °C for 2 h to allow for the formation of pre-immune complexes. Primary natural killer (NK) cells were obtained from buffy coats taken from healthy donors. Buffy coats were treated with RosetteSep human NK cell enrichment cocktail (StemCell) and then resuspended in RPMI supplemented with 10% FBS, 10 μg/ml of brefeldin A (MilliporeSigma), and a 1:10 dilution of GolgiStop (BD Biosciences). The cell suspension was added to the plates at a concentration of 25,000 cells/well and incubated at 37 °C for 5 h. Cells were fixed and permeabilized using the Fix and Perm Cell Permeabilization Kit (ThermoFisher Scientific), and then stained using anti-CD56, anti-CD3, anti-MIP-1β, and anti-IFN-γ antibodies (all BD Biosciences). Cells were run on an Intellicyt iQue Screener Plus and gates were drawn on singlet CD56^+^/CD3^-^ cells. Results were reported as the percentage of cells that were MIP-1β-positive.

### RNA-seq

Blood transcriptome analysis after vaccination was conducted by Next-Generation Sequencing (NGS). Whole-blood samples (250 μl) collected 18 days prior to vaccination and 1 and 3 days after vaccination were stored at −80 °C in DNA/RNA Shield (Zymo Research). Total RNA was isolated from whole blood samples using the Zymo Research Quick RNA Whole Blood Kit according to the manufacturer’s protocol. Sample libraries were globin-depleted and prepared using the SMART-Seq® stranded kit (Takara Bio) which includes poly(A) selection. Paired-end reads of 150 bp were sequenced using an Illumina HiSeq 4000.

For transcriptomics analyses, raw RNA-seq data were aligned to the *M. fascicularis* reference genome (Ensembl version 5.0) using STAR v2.6.1d^49^ and gene counts were computed using featureCounts v1.5.2.^50^ Differential expression analyses comparing post-vaccination transcript abundance to pre-vaccination abundance were performed using DESeq2 v1.28.0.^51^ Principal Component Analysis (PCA) was used to identify outliers among transcriptomic samples: if a sample’s first or second principal component value was more than three standard deviations from the corresponding principal component’s mean across samples, it was excluded from downstream analyses. Two samples were identified as outliers based on PCA: one from the control group collected 3 days after vaccination and one from the low-dose vaccinated group collected at the same timepoint.

Gene sets used to functionally profile groups of differentially-expressed transcripts were obtained through the tmod R package v0.46.2^52^ and the mSigDB Hallmark gene set.^53^ A total of 656 gene sets were used in the analysis. Homer Motif Analysis was used to identify known transcription factor binding motifs enriched in the promoter sequences of the human orthologs of genes determined to be differentially expressed (DEGs).^54^ STRING was used to identify known protein–protein interactions among DEGs using the high-confidence setting.^55^

### Vaccine efficacy and LASV challenge

On day 28 after vaccination, all animals were challenged by IM injection with 3.5 × 10^3^ PFU of LASV (lineage IV, Josiah strain). Virus challenge was performed in the ABSL-4 laboratories of UTMB. Viral stocks were prepared at UTMB using material originally derived from a 1976 Sierra Leone viral isolate collected from human serum of a 40-year-old male and passaged one time in Vero 76 cells and four times in Vero E6 cells. Macaques were monitored daily and scored for LASV disease progression using a humane endpoint arenavirus disease scoring sheet approved by the UTMB IACUC. The scoring changes measured from baseline included posture and activity level, attitude and behaviour, food intake, respiration, and disease manifestations, such as visible rash, haemorrhage, ecchymosis, or flushed skin. Animals were also monitored for central nervous system abnormalities. A score of ≥10 indicated that an animal met the criteria for euthanasia. Blood was collected via peripheral venipuncture on days 4, 7, 10, 11, 13, 15, 21 and 28 after LASV challenge for evaluation of blood chemistries and quantification of infectious LASV.

### Post-LASV challenge assays

To monitor LASV genomes in blood by RT-qPCR, RNA was isolated from whole blood using a viral RNA Mini Kit (Qiagen) using 100 μl of blood into 600 μl of viral lysis buffer AVL. Forward primer (5′-CACACAGGTGTCGTTGTTGA3′), reverse primer (5′-TTCAGTCCTCCTGACACTGC-3′) and probe 6-carboxyfluorescein (6FAM)-5′-CCCTCACTGTGCACTAATGGACTGC-3′-6 carboxytetramethylrhodamine (TAMRA) (Life Technologies) targeting the *N* gene (Genbank HQ688672) of LASV were used for RT-qPCR. LASV RNA was detected using the CFX96 detection system (Bio-Rad) with one-step probe RT-qPCR kits (Qiagen) with the following cycle conditions: 50 °C for 10 min, 95 °C for 10 s, and 40 cycles of 95 °C for 10 s and 59 °C for 30 s. Threshold cycle (CT) values representing LASV small (S) genomes were analysed with CFX Manager Software, and data are shown as genome equivalents (GEq). To create the GEq standard, RNA was extracted from LASV stocks, and the number of LASV S genome segments was calculated using Avogadro's number, the molecular weight of the LASV genome, and the percentage of the S genome segment to total viral RNA genomes present in the standard sample. The limit of detection was defined as 1 × 10^4^ GEq/ml.

Infectious LASV in collected macaque plasma was quantified by plaque assay as previously described.^33^ Briefly, serial 10-fold dilutions of plasma samples were allowed to infect Vero 76 monolayers in duplicate wells (200 μl/well). The limit of detection was defined as 25 PFU/ml of plasma.

Blood chemistries were analysed on collected serum using a Piccolo point-of-care analyser and Biochemistry Panel Plus analyser discs (Abaxis/Zoetis).

### Statistical analysis

Log-rank tests were used for Kaplan–Meier curves and Kruskal–Wallis tests with Dunn’s post-hoc multiple comparisons test^56^ for ELISA and ELISpot data to determine statistical differences. For statistical analysis of systems serology experiments, measurements were log-transformed and groupings were determined using the Benjamini-Hochberg procedure and Mann–Whitney U tests. All subjects were analysed in technical duplicates, and correlation between replicates was assessed prior to any subsequent analyses. Differences with p < 0.05 were considered significant. The analysis pipeline for systems serology is available at systemsseRology on GitHub. For analysis of aligned RNA-seq data, hypergeometric tests were used to assess gene set enrichment for DEGs. FDR-adjusted p-values [Benjamini-Hochberg method^57^] less than 0.05 were considered significant. All statistical calculations were carried out using Graphpad Prism or R.

### Role of the funding source

The funders did not have a role in the study design; in the collection, analysis, and interpretation of data; in the writing of the report; or in the decision to submit the paper for publication.

## Results

### Development of VSVΔG-LASV-GPC for human clinical trials

**Fig. 1:**
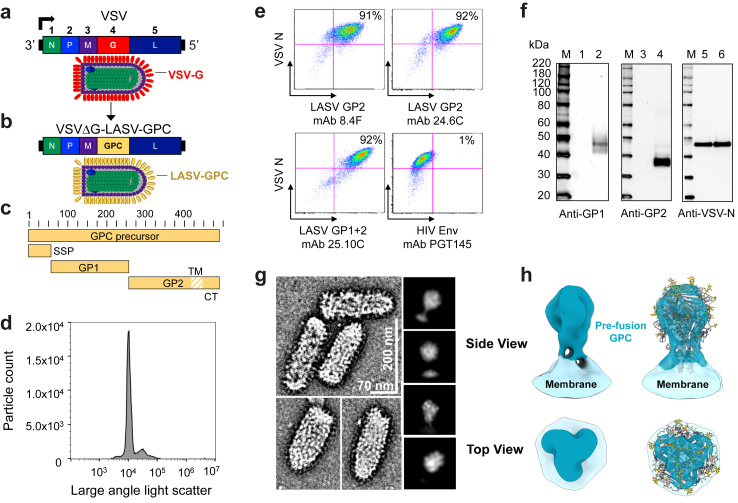
**VSVΔG-LASV-GPC chimeric virus vaccine.** (a) Illustration of the enveloped VSV particle and its (−)ssRNA genome. The genes encoding the N, P, M, G and L proteins are expressed from a single promoter at the 3’ end. Genes closest to the promoter are transcribed more abundantly.^58^ (b) Schematic of the replication-competent VSVΔG-LASV-GPC vaccine used in these studies.^25^^,^^33^ (c) Map of the GPC precursor protein and its proteolytic processing. SSP, stable signal peptide.^20^ (d) Nanoflow virometry was used to analyse purified VSVΔG-LASV-GPC vaccine material.^59^ Particle counts (ordinate) and large-angle light scatter (abscissa) are shown. (e) Expression and antigenicity of GPC on the cell surface was evaluated by flow cytometry using infected Vero cells and three different GPC-specific mAbs^40^ or a negative control antibody specific for HIV-1 Env (PGT-145^41^). Intracellular VSV N was stained with mAb 10G4.^42^ (f) Purified vaccine material was analysed by Western blot to detect GP1 (mAb 3.3B, lanes 1–2) and GP2 (mAb 22.5D, lanes 3–4).^40^ VSV N was detected in lanes 5 and 6 with a polyclonal rabbit antibody.^39^ Lanes 2, 4 and 6 contained VSVΔG-LASV-GPC; lanes 1, 3 and 5 contained VSVΔG-MARV-GP as a control.^38^ (g) Electron micrograph composition of negative-stained VSVΔG-LASV-GPC particles and reference-free 2D class averages of GPC coating the viral surface. This analysis was conducted with the vaccine material used in this study. (h) 3D reconstruction of GPC on the surface of VSVΔG-LASV-GPC with C3 symmetry applied (left panel), and with the full-length atomic model of GPC (PDB: 7PUY) docked into the reconstruction (right panel).

Like VSVΔG-ZEBOV-GP (ERVEBO),^24^^,^^27^ preclinical studies have shown that a single vaccination with VSVΔG-LASV-GPC (Fig. 1a–c) is consistently efficacious in macaques.^31^, ^32^, ^33^, ^34^ Therefore, our goal was to advance a VSVΔG-LASV-GPC vaccine suitable for use in people, confirm its efficacy in cynomolgus macaques that reproduce many aspects of human disease when infected with wild-type LASV,^60^^,^^61^ and study immune responses in more detail to identify biomarkers potentially associated with efficacy.

To generate a new recombinant virus with the documented history and genetic background needed to support development of a human vaccine, VSVΔG-LASV-GPC (Fig. 1a–c) expressing the Josiah lineage IV GPC^25^^,^^33^ was recovered from plasmid DNA and multiple clonal virus isolates were derived by three rounds of plaque isolation.^37^^,^^38^ During clonal virus isolation, multiple properties were monitored, including virus growth, GPC expression, and genomic nucleotide sequence. A lead candidate was selected whose genomic sequence was identical to the original genomic plasmid DNA,^25^ and this virus was then amplified to produce a preMVS that could be qualified for use in future manufacturing.

The VSVΔG-LASV-GPC preMVS was evaluated to ensure that it had properties needed to be advanced for human vaccination. Preclinical vaccine material, produced by infecting Vero cell cultures with the preMVS, was purified and concentrated by a process based on TFF. Analysis of purified virus by nanoflow virometry (Fig. 1d) showed that the vaccine material was composed of one predominant population. The particle count for the vaccine material was approximately 5.0 × 10^10^ particles per ml with the predominant peak accounting for 76% of the collected events. This corresponds to approximately 20 particles per PFU, assuming that the predominant peak was primarily composed of virion particles.

To investigate cell surface expression of LASV GPC, Vero cells were infected with VSVΔG-LASV-GPC and then analysed by flow cytometry (Fig. 1e). At about 48 h after infection (MOI 0.001), cells were harvested and incubated first with anti-GPC mAbs specific for different GPC epitopes or a negative control mAb specific for HIV-1 envelope (Env) glycoprotein (PGT-145) after which the cells were fixed and permeabilized for detection of intracellular VSV N. Over 90% of the cells stained with anti-GPC antibodies were positive for both intracellular VSV N and the cell-surface LASV glycoprotein. The GP2 subunit was detected by non-neutralizing mAbs 8.4F and 24.6C^40^ implying that some forms of the glycoprotein are not in a prefusion structure at this late stage of VSV infection. The anti-GPC mAb 25.10C, a potent nAb that binds to a structural epitope formed by the interaction of the GP1 and GP2 subunits assembled in a mature prefusion GPC^40^^,^^62^ also produced a strong signal indicating that native trimeric GPC was expressed following VSVΔG-LASV-GPC infection. Western blot analysis (Fig. 1f) also was conducted with purified VSVΔG-LASV-GPC (lanes 2, 4 and 6) and compared to purified VSVΔG-MARV-GP (lanes 1, 3 and 5). As expected, VSV N protein was detected in both samples (lanes 5 and 6), while LASV glycoprotein subunits GP1 and GP2 were only detected in lanes containing samples of VSVΔG-LASV-GPC (lanes 2 and 4, respectively). Together, the Western blot and flow cytometry analysis confirmed the correct processing and expression of the expected LASV GP1 and GP2.

Purified vaccine material was analysed by cryo-electron microscopy (Cryo-EM) to visualize VSV particles and GPC incorporated in the VSV envelope. Cryo-EM images of the bullet-shaped VSVΔG-LASV-GPC particles (Fig. 1g) indicated that individual virions have abundant GPC arrayed on their surface. Further 3D reconstructions provided evidence that GPC peplomers exist in a prefusion conformation (Fig. 1g–h). This is of particular importance, as glycoproteins densely arrayed on progeny VSV particles produced by replication *in vivo* likely are potent B cell immunogens.^63^

### VSVΔG-LASV-GPC efficacy in cynomolgus macaques after a single vaccination

**Fig. 2:**
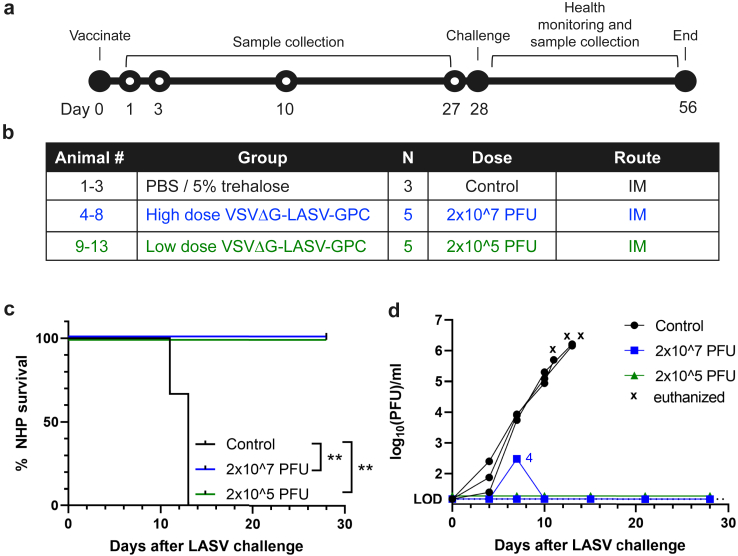
**VSVΔG-LASV-GPC vaccination and LASV challenge.** Cynomolgus macaques were divided into three groups including unvaccinated control macaques that were injected with buffer (n = 3; PBS containing 5% trehalose) and animals injected with 2 × 10^7^ PFU or 2 × 10^5^ PFU of VSVΔG-LASV-GPC (n = 5 per group). Vaccination was performed with a single IM injection, after which samples were collected through day 27 for immunological analysis. On day 28, animals were challenged with a single IM injection of 3.5 × 10^3^ PFU LASV (Josiah strain, lineage IV) and were monitored for an additional 28 days for signs of LASV disease. Animals exhibiting disease symptoms were euthanized based on predetermined health criteria. (a) Timeline of study activities. (b) Group descriptions. (c) Kaplan–Meier survival curves. One control animal became moribund on day 11 following challenge, the remaining two on day 13. The 10 animals vaccinated with VSVΔG-LASV-GPC survived until the completion of the study. ∗∗, p < 0.01 by log-rank test. (d) Infectious LASV in blood of challenged animals was quantified by plaque assay. LOD, limit of detection.

Features of human LASV disease are observed in infected cynomolgus macaques.^60^^,^^61^ Thus, macaques are a valuable model for evaluating LASV vaccine candidates and were used here to assess VSVΔG-LASV-GPC efficacy. Two groups of five cynomolgus macaques (two males and three females per group) were vaccinated once by IM injection with 2 × 10^7^ or 2 × 10^5^ PFU of VSVΔG-LASV-GPC (lineage IV, Josiah), while three control animals (one female and two males) were injected with vaccine diluent (Fig. 2a–b). The higher vaccine dose of 2 × 10^7^ PFU was selected because similar doses have been used previously to vaccinate both macaques and people with VSVΔG chimeras.^23^^,^^24^^,^^33^ No observable adverse reactions were induced by either vaccine dose. RNA containing a VSV N target sequence was undetectable by RT-qPCR in blood samples collected at 1, 3 and 10 days after vaccination (Supplementary Table S1), consistent with data from an earlier study in which VSVΔG-LASV-GPC did not produce detectable viraemia.^33^

Animals were challenged 28 days after vaccination (Fig. 2a) by IM injection with an infectious dose of 3.2 × 10^3^ PFU of LASV (lineage IV, Josiah strain, low-passage stock). The three mock-vaccinated control animals developed clinical signs of LASV disease that mandated euthanasia at 11–13 days after challenge, whereas 10 of 10 vaccinated animals remained healthy to the end of the study at day 56 (28 days after LASV infection, Fig. 2c). When LASV viraemia was evaluated by plaque assay, unvaccinated control macaques were found to have developed substantial titres of infectious LASV as early as day 4 after challenge and exceeding 1 × 10^6^ PFU per ml of plasma at the time of euthanasia (Fig. 2d). Conversely, no infectious LASV was detectable in macaques vaccinated with 2 × 10^5^ PFU, while a single animal vaccinated with 2 × 10^7^ PFU developed low-level (∼300 PFU per ml) transient viraemia detected on day 7 (Fig. 2d). Analysis of LASV RNA in whole blood showed that genome copies were detectable on days 7 and 10 in the same animal, but not on later days 15, 21 or 28 after infection (Supplementary Fig. S1). LASV RNA also was detected in one animal in the low-dose group on day 7, although no infectious LASV could be detected in blood from this animal (Supplementary Fig. S1). The three control animals displayed high RNA copy numbers (Supplementary Fig. S1) consistent with LASV viraemia (Fig. 2d).

**Fig. 3:**
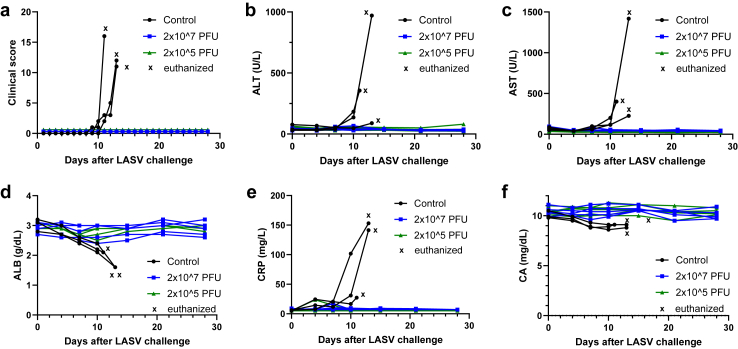
**Health monitoring and blood chemistry following LASV challenge.** (a) Clinical observation scores through day 28 after LASV challenge. (b–f) Key analytes in the blood of animals were quantified from the day of challenge to the end of the study: (b) alanine aminotransferase (ALT); (c) aspartate aminotransferase (AST); (d) albumin (ALB); (e) C-reactive protein (CRP); and (f) calcium (CA). n = 3–5/group.

Clinical observations (Fig. 3a) reflected development of viraemia (Fig. 2d). Control animals developed clinical signs within 9 days after LASV challenge that increased in severity up to euthanasia (Fig. 3a and Supplementary Fig. S2). In addition, control animals exhibited neurological signs, changes in respiration, loss of appetite, and changes in general activity and appearance (Supplementary Fig. S2). Blood chemistry values in unvaccinated animals indicated abnormal liver function (increased alanine aminotransferase [ALT] and aspartate aminotransferase [AST], decreased albumin [ALB]), as well as increased acute phase inflammatory responses (increased C-reactive protein [CRP]) and electrolyte imbalance (decreased calcium; CA) following LASV infection (Fig. 3b–f). The 10 vaccinated animals did not develop any clinical signs of disease or changes in blood chemistry (Fig. 3). Together, these data demonstrate that the VSVΔG-LASV-GPC vaccine prepared for clinical development protected 10 of 10 vaccinated macaques from developing clinical signs of Lassa virus disease.

### VSVΔG-LASV-GPC humoural and cellular immunogenicity

**Fig. 4:**
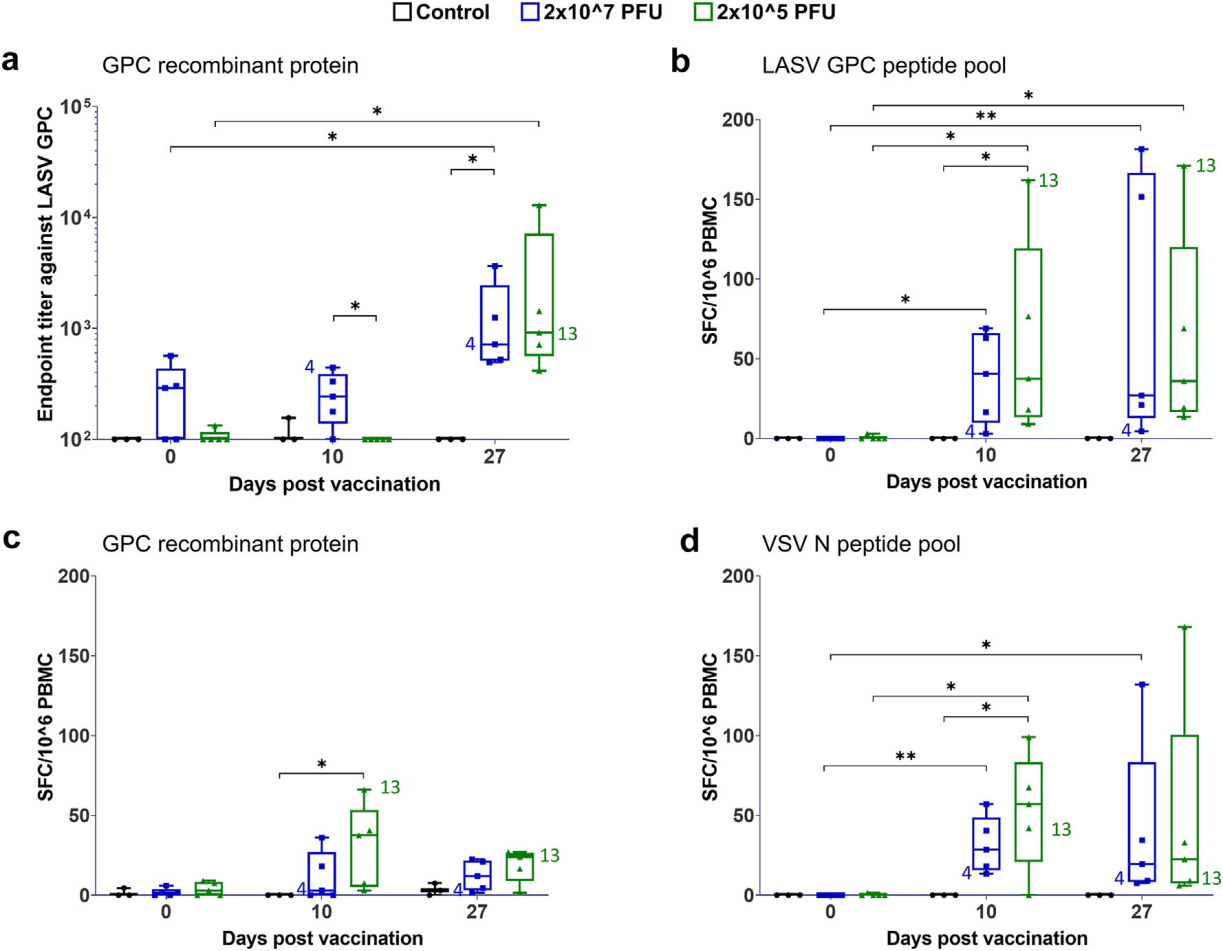
**Quantification of adaptive immune responses.** (a) Seroconversion was monitored by direct ELISA using plates coated with recombinant, covalently linked GP1–GP2 (GPC). (b–d) The presence of peripheral blood T cells specific for LASV GPC or VSV N was quantified by IFN-γ ELISpot assay. PBMCs from vaccinated animals were stimulated *in vitro* with (b) overlapping peptides spanning the complete sequence of GPC; (c) the same recombinant linked GP1–GP2 protein (GPC) used for ELISA in (a); or (d) overlapping peptides spanning the complete sequence of VSV N. Results are expressed as spot-forming cells (SFC) per one million PBMCs. Box plots indicate the interquartile range and median. Data from viraemic/RNAemic animal are indicated by their identifier (4 and 13). ∗∗, p < 0.01; ∗, p < 0.05 by Kruskal–Wallis test with Dunn’s post-hoc multiple comparisons test. n = 3–5/group.

To develop a profile of immune responses associated with efficacy, humoural immunity was evaluated first by measurement of anti-GPC IgG titres. Serum samples collected on the day of vaccination and after vaccination on days 10 and 27 were analysed by ELISA (Fig. 4a). A soluble form of a covalently linked GP1–GP2 (Josiah, lineage IV;^17^) was used as the antigen. By day 10 after vaccination, anti-GPC antibodies were not yet detectable in animals vaccinated with the lower dose (2 × 10^5^ PFU) while macaques vaccinated with 2 × 10^7^ PFU did have modest anti-GPC IgG titres at this time point; but as several of these animals had a comparatively high level of background signal at baseline, it is unclear whether the titres at day 10 reflect an increase due to vaccination. By day 27 after vaccination, however, 100% seroconversion was observed in both vaccine groups with median endpoint titres of 719 (mean 1330; range 496–3661) and 915 (mean 3274; range 415–12,897), respectively.

To ascertain the prevalence of vaccine-induced T cells, PBMCs were stimulated overnight with overlapping peptides spanning GPC (Fig. 4b), the same soluble recombinant GP1–GP2 fusion protein used in ELISAs (Fig. 4c), or overlapping VSV N peptides (Fig. 4d), and IFN-γ-secreting cells were quantified by ELISpot. Vaccination with either dose induced detectable GPC-specific T cell responses in 90% of animals, although antigen-specific T cell frequencies varied within each group (Fig. 4b–c). IFN-γ responses stimulated by the VSV N peptide pool were of comparable magnitude and variability to the GPC responses (Fig. 4d). These data demonstrate that vaccination with VSVΔG-LASV-GPC induced both humoural and cellular immune responses with nearly 100% response rates detected as early as day 10 after vaccination.

### Evaluation of neutralizing antibodies

**Fig. 5:**
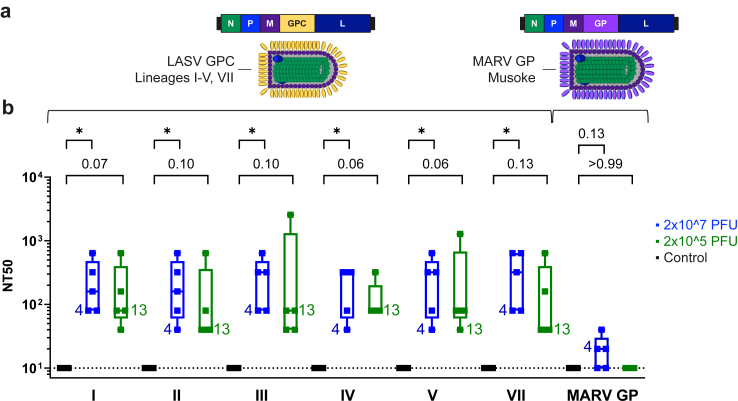
**Quantification of neutralizing serum antibodies.** VSVΔG-LASV-GPC chimeras expressing GPC from LASV lineages I–V and VII or MARV GP (Musoke; VSVΔG-MARV-GP) were used in a PRNT to quantify serum nAbs. Genbank accession numbers for the GPC sequences used in development of the VSVΔG-LASV-GPC chimeras are included in Supplementary Table S2. The ability of serum from day 27 after vaccination to neutralize these virions was evaluated. (a) Schematic representing the replication-competent viruses used in this assay. (b) Serum neutralizing titres. Values plotted are the serum dilutions that reduced the number of plaques by 50% (NT50). Boxplots indicate the interquartile range and median. Data from viraemic/RNAemic animals are indicated by their identifier (4 and 13). This assay may provide a more sensitive assessment of neutralization than single-cycle infection (see Discussion). Results from intraclade Kruskal–Wallis test with Dunn’s post-hoc multiple comparisons test are indicated. ∗, p < 0.05. n = 3–5/group.

We next investigated the ability of serum from vaccinated and control animals to neutralize virus using a PRNT based on neutralization of VSVΔG-LASV-GPC. To assess the breadth of neutralization, replication-competent VSVΔG-LASV-GPC chimeras were generated that expressed GPC from LASV lineages I–V and VII (Supplementary Table S2) to represent strains from diverse geographical regions.^1^ Serum collected 27 days after vaccination on the day before LASV challenge was found to contain detectable neutralization activity against homologous lineage IV GPC (Fig. 5). Animals vaccinated with 2 × 10^7^ PFU displayed a median 50% neutralizing titre (NT50) of 320 (mean 216; range 40–320), while samples from the 2 × 10^5^ PFU group trended lower at a median NT50 of 80 (mean 128; range 80–320). Notably, when neutralization activity against VSVΔG-LASV-GPC encoding heterologous GPC from lineage I, II, III, V and VII was assessed, neutralization titres were like those detected with homologous lineage IV (Fig. 5b). No meaningful neutralization of the control virus, VSVΔG-MARV-GP,^38^ was detected in serum from any animals vaccinated with VSVΔG-LASV-GPC. Taken together, the neutralization titres indicated that a single dose of the VSVΔG-LASV-GPC lineage IV vaccine induced nAbs effective against GPC from a broad range of LASV lineages.

### Evaluation of Fc-mediated antibody effector functions

In addition to direct neutralization, other antibody-mediated functions can contribute to protective immunity. Non-neutralizing effector functions are typically mediated by interactions of the antibody constant domain (also known as Fragment crystallizable domain, Fc domain) with innate immunologic effector cells through Fc gamma receptors (FcγRs or C-type lectin receptors) or with complement proteins.^64^, ^65^, ^66^ Interestingly, in the case of SARS-CoV-2, direct neutralization activity elicited by vaccination may diminish as variants emerge with mutations in the receptor-binding domain, but antibodies that can direct non-neutralizing effector functions through Fc receptors may contribute to continued prevention of serious illness.^67^ Moreover, nAbs can have their neutralization function potentiated through Fc modifications, indicating that both Fab and Fc domains contribute to optimized antibody function.^66^^,^^68^ Therefore, we evaluated the potential for anti-GPC serum antibodies to promote Fc-directed functions.

Antibody isotypes and subclasses are important determinants of Fc-directed effector functions. Therefore, we first analysed the isotype and Fc profile of GPC-specific serum antibodies (Supplementary Fig. S3). The antigens used in the assay included the GP1–GP2 fusion protein (GP-link) identical to the ELISA antigen (Fig. 4a) and a GP1–GP2 protein complex (GP-prefusion) containing strategic disulfide bonds to stabilize a more native-like prefusion trimeric structure.^17^ Lineage IV GPC homologous to the vaccine was used as well as lineage II GPC to represent a divergent LASV that circulates in parts of Western Africa^1^ where a phase 2 clinical trial has begun (ClinicalTrials.gov: NCT05868733 and^15^^,^^69^). When sera from day 10 and day 27 were analysed, most animals per vaccine group demonstrated increased signals for IgG1 binding to both forms of lineage II and IV antigens compared to day 0. IgG2, IgG3, IgA or IgM specific for GPC were also detectable in both groups, though less strongly. Interestingly, IgM responses were largely limited to the low-dose group (Supplementary Fig. S3e).

**Fig. 6:**
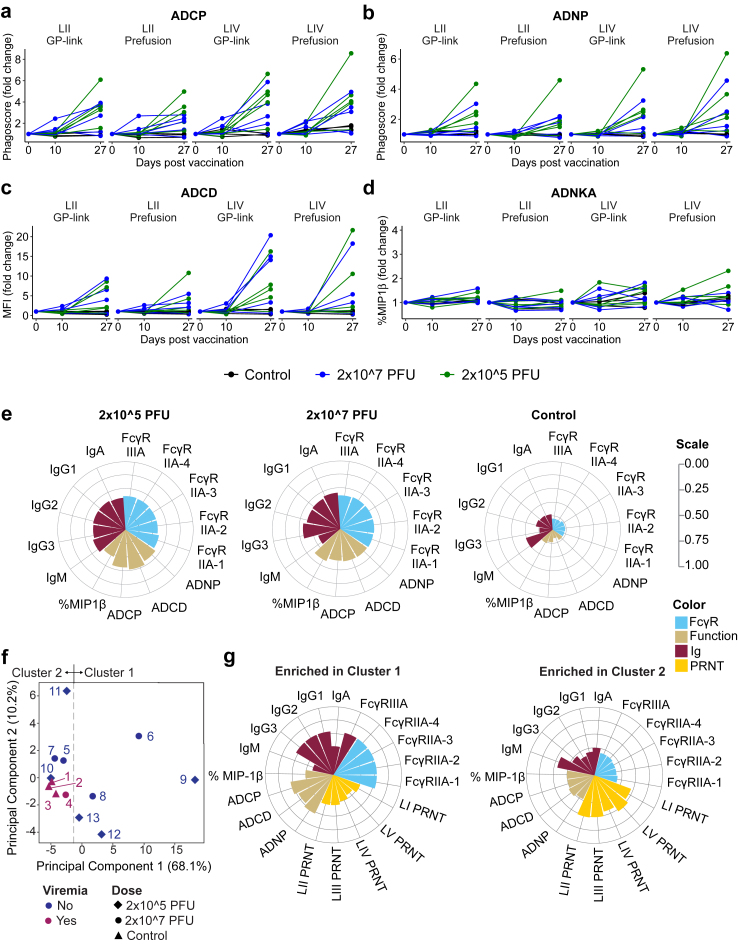
**Effector properties of vaccine-elicited antibodies in serum.** Serum harvested on day 0, 10 and 27 after vaccination was used to quantify different non-neutralizing effector functions of vaccine-induced serum antibodies. (a) Antibody-dependent cellular phagocytosis (ADCP); (b) antibody-dependent neutrophil phagocytosis (ADNP); (c) antibody-dependent complement deposition (ADCD); and (d) antibody-dependent NK-cell activation as measured by MIP-1β expression (ADNKA). Assays were performed with lineage II or lineage IV GP antigens including a soluble GP that adopts a native-like prefusion conformation (GP prefusion) or a soluble GP1–GP2 fusion protein (GP-link). (e) Polar plots of the median percentile rank of each antibody function, isotype, and FcγR binding titre against LIV GP-link at day 27. Legend is shared with panel (g) and shown on the right indicating rank score (upper right) and colour scheme (lower right). (f) A PCA was built using all available data on antibody features. Colouring corresponds to the detection of viraemia (*cf.*Fig. 2d). (g) Polar plots of the median percentile rank for all antibody features for animals binned by segregation along PC2 or PC1, as indicated. The legend is shared with (e). n = 3–5/group.

Compared to antibodies in preimmune sera, serum antibodies complexed with bead-conjugated GPC protein were shown to bind to soluble FcγRIIA-1, FcγRIIA-2, FcγRIIA-3, FcγRIIA-4, and FcγRIIIA after vaccination with VSVΔG-LASV-GPC (Supplementary Fig. S3f–j) and this likely was driven largely by IgG1. The Fc domain of IgG1 can bind FcγRII and FcγRIII to promote phagocytosis, antibody-dependent cell cytotoxicity (ADCC), and cell lysis through complement.^66^ Therefore, we conducted assays to analyse antibody-dependent cellular phagocytosis (ADCP; Fig. 6a), neutrophil phagocytosis (ADNP; Fig. 6b), complement deposition (ADCD; Fig. 6c) and NK cell activation (ADNKA; Fig. 6d). Consistent with anti-GPC binding titres (Fig. 4a), signals at day 10 in these assays generally were low. However, serum from most animals on day 27 directed at least some ADCP, ADNP, ADCD and/or ADNKA activity above the baseline of serum from day 0 in both vaccine groups, with generally slightly higher responses to homologous lineage IV GPC than to the heterologous lineage II GPC. ADCP, ADNP and ADCD responses were induced with approximately equal magnitude by GP-link and GP-prefusion. ADNKA responses, as measured by the increase in MIP-1β expression, were detectable but generally muted (Fig. 6d).

To better visualize the extent of these Fc-mediated antibody functions, results produced with the lineage IV GP1–GP2 (GP-link) fusion protein by serum from day 27 were summarized in polar plots of the mean percentile rank for each antibody feature and function (Fig. 6e). Individual antibody isotypes, subclasses, Fc-binding antibodies, and antibody-effector functions were standardized via z-scoring to enable cross-methodology comparisons as described previously.^70^ These plots emphasized the robustness of the observed responses relative to background activity of serum from control animals, as well as the high similarity in the nature and quality of the antibody effector functions between the two dose groups. Together, these results indicate that VSVΔG-LASV-GPC vaccination elicited anti-GPC polyclonal antibodies capable of directing cellular phagocytosis, limited NK-cell activation, and complement deposition, all of which may contribute to protection.

To evaluate which features of the humoural response were most strongly associated with protection from LASV disease, our collective antibody data were integrated and assessed with a principal component analysis (PCA; Fig. 6f). We focused on antibody data given the extent of humoural immune response characterization and that anti-GPC serum antibodies and nAbs were detected in the 10 vaccinated animals (Fig. 4, Fig. 5). The PCA showed that the three non-vaccinated control animals grouped together and with macaque #4, which was the only vaccinated animal that had developed transient, low-level viraemia (Fig. 2d). This grouping of animals also included one macaque from the low-dose group, macaque #10 (Fig. 6f), which had not exhibited viraemia or clinical signs of disease (Fig. 2, Fig. 3). In general, most vaccinated animals separated from the control animals along either principal component (PC) 1 or PC2, with only one animal from the high-dose group, macaque #6, displaying an intermediate phenotype with features of both PC1 and PC2 (Fig. 6f). We next binned the animals based on their primary segregation along either PC1 (Cluster 1 in Fig. 6f) or PC2 (Cluster 2) and plotted all the analysed antibody features on polar plots (Fig. 6g). The graphs illustrated that nAb titres active against the GPC from different lineages were the most prominent feature in animals in Cluster 2, while Fc-dependent functions and titres of anti-GPC antibodies of different IgG subtypes were more predominant in the Cluster 1 profile. This analysis is consistent with VSVΔG-LASV-GPC inducing polyclonal antibodies that may contribute to protection through virus-neutralizing antibodies or through Fc-directed effector functions and that composition of functional antibodies varies between animals. To determine whether sex was a major determinant in the antibody response to vaccination with VSVΔG-LASV-GPC, we overlaid the PCA with the sex of each animal (Supplementary Fig. S4a). Animals of different sex were found in Clusters 1 and 2, suggesting that this was not a major determinant of the nature of the observed antibody responses.

Integration of the results from these metrics of adaptive immunity also reflected a diversity of responses, with animals differing in which response types were most prominent (Supplementary Fig. S4b and Table S3). Macaque #4, the only animal that had developed transient viraemia (while remaining fully protected from clinical signs of disease), exhibited overall less pronounced responses but was not a clear outlier, for instance compared to animal #10, consistent with the PCA analysis in Fig. 6f. Animal #13, which displayed a transient spike LASV RNA but no detectable live virus, exhibited relatively high titres of anti-GPC IgM but otherwise had highly similar immune responses to the other vaccinated animals.

### Early immune response to vaccination

**Fig. 7:**
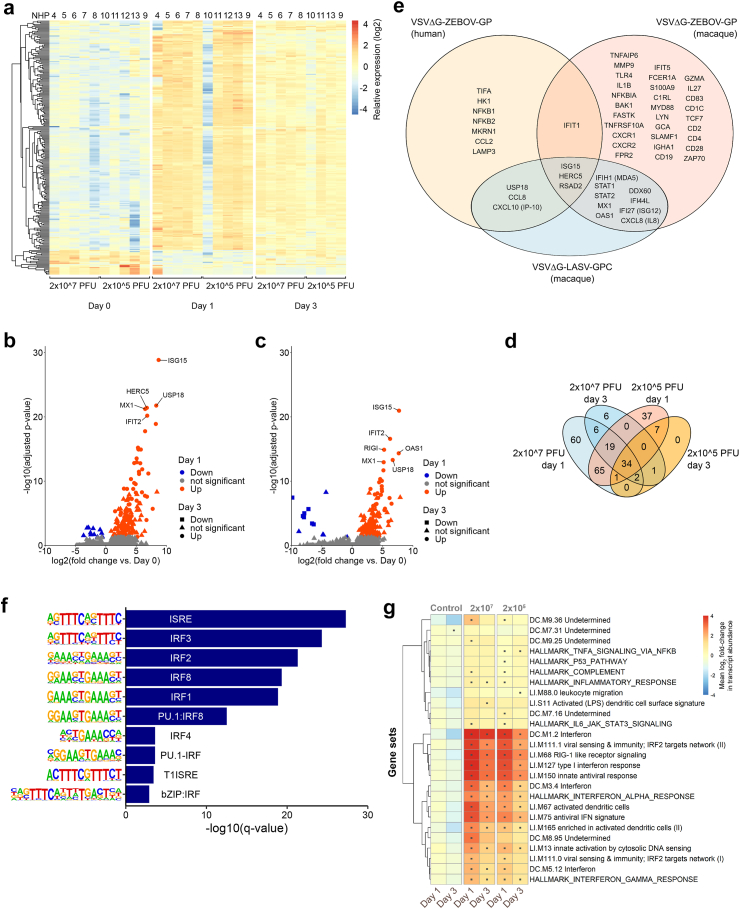
**Effect of IM injection with VSVΔG-LASV-GPC on the whole-blood transcriptome.** Gene expression prior to and on days 1 and 3 after vaccination was analysed by RNA-seq on whole-blood samples from the high-dose (2 × 10ˆ7 PFU) and low-dose (2 × 10ˆ5 PFU) groups. (a) Heatmap showing normalized expression values of all differentially expressed genes (DEGs) with unsupervised hierarchical clustering. (b and c) Volcano plots showing changes in expression and the associated statistical significance in the (b) high-dose and (c) low-dose group, respectively, on day 1 after vaccination compared to baseline. Symbol shape indicates whether a given gene was differentially expressed on day 3 after vaccination. (d) Venn diagram showing the overlap of DEGs between the two time points and the two dose groups. (e) Venn diagram of the overlap between VSVΔG-LASV-GPC-induced DEGs and a framework of representative genes identified previously as differentially expressed following vaccination with VSVΔG-ZEBOV-GP in macaques and humans.^35^^,^^36^^,^^72^ (f) Transcription factor motifs enriched in the promoter region of VSVΔG-LASV-GPC-induced DEGs, identified by Homer.^54^ (g) Heatmap showing mean log_2_ fold-changes in expression of genes within gene sets enriched for VSVΔG-LASV-GPC-induced DEGs. Gene sets in the heatmap include those that showed significant enrichment for DEGs in at least one study group at one or both post-vaccination timepoints. Dots in heatmap cells indicate significant enrichment for DEGs. “DC”, “LI” and “HALLMARK” prefixes on gene sets indicate the published collection from which they originated,^53^^,^^73^^,^^74^ respectively. n = 3–5/group.

We also investigated the blood transcriptome early after vaccination because VSV infection and replication is expected to play a prominent role in inducing early innate immune responses that promote development of protective adaptive immunity.^71^ RNA extracted from blood prior to and at days 1 and 3 after vaccination was analysed using RNA sequencing (RNA-seq). There were no major perturbations to the overall transcriptome, suggesting that vaccination, as expected, did not result in a large-scale change in the composition of blood cell populations (Supplementary Fig. S5). Instead, high- and low-dose vaccination both resulted in a strong response that was focused on 238 genes, with expression changes for some genes exceeding 400–fold compared to pre-vaccination samples (Fig. 7a). The majority of these DEGs were up-regulated upon vaccination, an effect that was markedly diminished by day 3 (Fig. 7b high-dose and 7c low-dose). Importantly, there was a high degree of overlap between genes whose expression was altered by high-dose and low-dose vaccination (Fig. 7d and Supplementary Table S4). *ISG15, USP18, IFIT2* and *MX1* were among the six most significantly altered genes in each dose group (Fig. 7b–c). There was a noticeable similarity between the DEGs identified in this study and sets of representative DEGs previously identified after vaccination with VSVΔG-ZEBOV-GP in both humans and macaques (Fig. 7e). Of note, one animal in the low-dose group, macaque #10, did not exhibit the same gene expression changes on day 1 as the other nine vaccinees but was still fully protected from lethal LASV challenge (Fig. 7a). Interestingly, this was the same animal whose integrated antibody responses clustered with the unvaccinated control group in the PCA (Fig. 6f). Although this is just one animal, the results might suggest that the full breadth and magnitude of early responses observed in most animals may not be strictly required for full protection.

To determine whether the DEGs identified in our study shared common transcriptional control pathways, we used Homer^54^ to perform a search for known transcriptional control elements regulating the DEGs. Indeed, Homer analysis identified 10 control elements exhibiting significant enrichment across the DEGs that were directly related to IFN-I signalling, consistent with a robust innate immune response to a replication-competent RNA virus (Fig. 7f). Similarly, an analysis of known interactions between the proteins encoded by the DEGs using STRING^55^ identified a tight, high-confidence network centred around regulators and effectors of IFN-I signalling (Supplementary Fig. S6). We also functionally profiled the DEGs by testing their enrichment for previously defined modules of coordinately-expressed genes. This approach identified 26 gene sets with significant enrichment, most of which were related to innate viral sensing and IFN responses (Fig. 7g). It was notable that multiple gene sets defined previously by Li et al.^73^ were affected by VSVΔG-LASV-GPC early after administration to macaques, and that a sizeable number of these modules were also enriched for the representative DEGs associated with VSVΔG-ZEBOV-GP vaccination (Supplementary Fig. S7a,^28^^,^^36^^,^^72^). This provides further evidence that both vaccines may elicit shared blood transcriptome responses even though viral infection and replication is mediated through differing VSV-expressed glycoproteins.

To determine whether the early transcriptomic responses to vaccination differed between male and female animals, we determined genes that were differentially expressed between both sexes in each dose group at either day 1 or 3 and looked for overlap between these sex-based DEGs and the vaccine-induced DEGs (Supplementary Fig. S7b). As expected, many genes were differentially expressed between male and female animals (2609 in the low-dose group, 1729 in the high-dose group), but only very few of those were also among the vaccine-induced DEGs (26 in the low-dose group, 6 in the high-dose group). This limited overlap was not statistically significant except for the low-dose group on Day 1. In that group and at that time point, there were 23 genes that did overlap between the vaccine-induced DEGs and sex-based DEGs, including several IFN-regulated genes. However, this difference was not replicated in the high-dose group, nor were there any differences between male and female animals in antibody responses or protective efficacy (Fig. 2, Fig. 3, and Supplementary Fig. S4), suggesting that any sex-specific differences are not robust or functionally relevant for this vaccine. Thus, vaccination with VSVΔG-LASV-GPC induces transcriptomic changes in blood cells that are dominated by type-I IFN signalling and are similar to the changes observed after vaccination with VSVΔG-ZEBOV-GP.

## Discussion

Our results showed that a single dose of 2 × 10^7^ or 2 × 10^5^ PFU of the VSVΔG-LASV-GPC human vaccine candidate protected 10 of 10 cynomolgus macaques against challenge with a lethal dose of homologous lineage IV LASV (Fig. 2). This result agrees with earlier preclinical efficacy studies conducted with experimental VSVΔG-LASV-GPC vaccines based on the same recombinant virus design.^31^, ^32^, ^33^, ^34^ A VSVΔG-based LASV vaccine modified to further attenuate its replicative capacity (rVSV-N4ΔG-LASVGPC) also has been shown to protect macaques vaccinated using a prime-boost vaccination regimen^75^ providing additional evidence that delivery of functional GPC with replicating VSVΔG-based vaccine is effective. Our work extends earlier research studies by showing that a plaque-purified VSVΔG-LASV-GPC qualified for human vaccination is efficacious (Fig. 2), a 100–fold lower dose of this vaccine is equally protective (Fig. 2), and vaccination elicited nAbs active against GPCs from multiple LASV lineages (Fig. 5). In addition to protecting from disease, vaccination prevented development of clinical pathologies associated with LASV infection such as elevated clinical scores or changes in blood chemistry (Fig. 3, Supplementary Fig. S1 and S2), and just one of the 10 vaccinated animals had detectable titres of infectious LASV in peripheral blood after challenge and the quantities were low and transient (∼300 PFU/ml; Fig. 2d). Our results differ from the first preclinical efficacy study conducted with a research-grade VSVΔG-LASV-GPC vaccine,^33^ as vaccinated animals developed transient LASV viraemia of up to 10^4^ PFU per ml of blood although they were protected from serious illness like observed here. It is unclear whether this difference in viraemia was due to the 3-fold higher challenge virus dose used in the prior study,^33^ the immunogenicity of the clonal VSVΔG-LASV-GPC isolate developed here, vaccination with vaccine material of higher purity, or some combination of these factors. The overall strong record of preclinical efficacy and positive interim results emerging from the phase 1 clinical trials (ClinicalTrials.gov NCT04794218 and IAVI, data not shown) have provided a strong rationale for further clinical development of VSVΔG-LASV-GPC, and a phase 2 clinical trial has been initiated in West Africa (ClinicalTrials.gov: NCT05868733 and^15^^,^^69^).

Immune responses that contribute to protection from LASV disease are not entirely understood. Recovery of LF patients is correlated with strong activation of antigen-specific peripheral blood T cells suggesting that cellular immunity plays a prominent role in natural acquired control and clearance of LASV infection.^6^ In contrast, nAbs develop slowly in LF patients and may not increase substantially until late convalescence.^6^ The delayed nAb response may be due to humoural immunity dysregulation during acute LASV infection and further suggests that antibody-mediated neutralization does not make a substantial contribution to viral clearance in unvaccinated individuals.^6^ Like these observations in infected people, recovery of macaques from experimental LASV infection has been shown to correlate with development of rapid and potent innate and cellular immunity, provided it did not lead to an excessive inflammatory response.^8^^,^^9^ Our data show that prophylactic vaccination with VSVΔG-LASV-GPC induces a different profile of immunity than develops during recovery from LF. We found that vaccination elicited detectable but relatively low frequencies of GPC-specific peripheral blood T cells secreting IFN-γ, whereas the 10 vaccinated animals developed anti-GPC serum antibodies and nAbs (Fig. 4). These data suggest that antibodies developed by vaccinated macaques played a role in protection from LASV disease, but they do not exclude the possibility of significant contributions from T cells, as most of the vaccinated macaques did develop detectable peripheral blood T cells specific for GPC (Fig. 4). A more extensive immunologic assessment of cellular immunity, including evaluation of T cell polyfunctionality and tissue resident responses,^76^ may better define contributing cellular immune responses.

It was not unexpected to find that live VSVΔG-LASV-GPC vaccination produced a different immune response profile than a LASV infection, because the two viruses have substantially different properties and effects on the immune system. The properties of VSV factored into our decision to conduct a relatively detailed analysis of antibodies elicited by vaccination, as VSV is acutely cytopathic, which favours development of B cell responses, while arenavirus infection causes less cytotoxicity that tends to promote T cell responses.^77^ Furthermore, LASV infection of dendritic cells and macrophages is known to impair their activation and ability to properly execute their roles in cytokine expression and antigen presentation^78^, ^79^, ^80^, ^81^ while VSV infection of these cells leads to immune activation.^82^, ^83^, ^84^ Thus, immune responses develop under much different conditions during a mild infection caused by VSVΔG-LASV-GPC compared to a LASV infection in a patient with LF, and this likely is an important factor in VSVΔG-LASV-GPC vaccination eliciting protective antibodies. In fact, the self-limiting VSV infection that occurs in a natural host like horses rapidly induces neutralizing serum antibodies and their development is correlated with resolution of vesicular lesions, although T cells also infiltrate the lesion indicating that they play some role in viral clearance.^85^ Some participation by T cells in clearing a natural VSV infection is consistent with our ELISpot data, which did detect a measurable frequency of GPC- and VSV-N-specific peripheral blood T cells. Overall, the suggestion that VSV infection is effective in eliciting protective antibodies against viral surface glycoproteins while eliciting a modest T cell response^86^, ^87^, ^88^ is supported by clinical research with VSVΔG-ZEBOV-GP, which indicates that anti-EBOV GP serum antibodies and nAbs are associated with protection^30^^,^^89^ and that vaccination elicits nAbs like those isolated from patients that recovered from EBOV infection.^90^

Our analysis indicated that VSVΔG-LASV-GPC elicited serum nAbs in the 10 vaccinated macaques (Fig. 5). The nAb titres in Fig. 5 were higher than those reported in the companion manuscript (Enriquez, Hastie et al., *submitted companion manuscript* *published in this issue*) and this was probably due to use of different neutralization assays and supporting methods. The data in Fig. 5 were generated with a PRNT based on replication-competent VSVΔG-LASV-GPC expressing GPC from different lineages while in the companion manuscript neutralization activity was assessed using a GPC-pseudotyped single-cycle VSV encoding GFP (VSVΔG-GFP) and quantifying fluorescent infected cells. The PRNT and assays based on single-cycle VSVΔG pseudovirions have been used before for detecting anti-Ebola virus serum neutralization activity and they were found to be useful surrogates for neutralization of authentic EBOV.^91^ Moreover, assays based on VSVΔG-ZEBOV-GP PRNT or single-cycle VSVΔG-Luciferase pseudovirions have been used in VSVΔG-ZEBOV-GP clinical trials.^92^, ^93^, ^94^ Thus, we hypothesize that differences in assay sensitivity for detection of anti-GPC nAbs across our studies was likely due to supporting procedures such as purification of the viruses used in the assays. VSVΔG-LASV-GPC virions used in the PRNT were purified by ultracentrifugation through sucrose cushions to remove contaminants from lysed cells, whereas VSVΔG-GFP pseudovirions harvested in culture medium from infected cells were clarified simply by centrifugation before virus stocks were frozen. Potentially, the purity of virions might affect antibody neutralization and assay sensitivity.

Lending support to the hypothesis that nAbs induced by VSVΔG-LASV-GPC vaccination contribute to protection, antibody neutralization activity has been associated with protection before. For example, convalescent serum from a person who had recovered from LF or serum from recovered macaques was shown to protect naïve macaques from LASV disease if the transferred sera had sufficient neutralizing activity.^95^ Although this study provided convincing evidence to show that passively transferred serum antibodies can protect, it has been challenging to specify the quantity of neutralization activity needed for therapeutic value, as some clinical trials have failed to detect benefit from transfer of immune sera.^4^ Assigning a protective threshold nAb titre may be complicated by protection being due to a combination of diverse effector functions directed by neutralizing and non-neutralizing antibodies, with their relative contribution varying in different individuals as our data suggest in Fig. 6. Studies with neutralizing anti-GPC mAbs with known epitope specificity provide clearer demonstration that nAbs can protect, as mAb infusion has been shown to be efficacious in guinea pigs and macaques when administered immediately following LASV challenge, and notably, when administered 8 days following virus exposure.^10^, ^11^, ^12^^,^^96^

We also showed that vaccination with VSVΔG-LASV-GPC expressing lineage IV GPC elicited nAbs active against GPC from lineages I–V and VII (Fig. 5). Because nAbs are a prominent surrogate for protective immunity induced by multiple successful viral vaccines,^21^ this finding suggests that the VSVΔG-LASV-GPC human vaccine candidate has the potential to provide broad protection against LASV from different geographical regions. In agreement with observing broad neutralization activity, serum antibodies from animals vaccinated in this study were found to target key conserved structures on the LASV GPC trimer (Enriquez, Hastie et al., unpublished, *submitted companion manuscript* *published in this issue*).

The possibility that native or native-like trimeric lineage IV GPC can stimulate B cells that produce broadly active nAbs also is supported by studies with a range of other experimental LASV vaccines.^18^^,^^97^, ^98^, ^99^ For example, a single vaccination with a measles virus vector-based vaccine encoding the native LASV lineage IV GPC and nucleoprotein protected macaques from LASV challenge.^98^ Although serum nAbs were undetectable after vaccination, broadly active nAbs developed soon after infection with LASV suggesting that vaccination established anti-GPC memory B cells that rapidly responded to LASV infection. Cross-neutralizing antibodies also have been elicited in rabbits vaccinated four times with VLPs that displayed native lineage IV GPC.^99^ Additionally, structural data from analysis of the native-like lineage IV GPC (GPCysR4^17^) was applied to development of two experimental vaccines that elicited broadly active nAbs in small animals. In one study, native-like trimers arrayed on self-assembling nanoparticles were shown to elicit broad serum neutralization activity in rabbits after three vaccinations.^97^ In the second, a trimerization domain was used to further stabilize lineage IV native-like trimer immunogens and guinea pigs vaccinated multiple times with the soluble trimeric form followed by boosting with a nanoparticle-based vaccine developed cross-neutralizing antibodies. Collectively, these data indicate that immunogens based on lineage IV GPC can elicit cross-reactive nAbs, and that delivery with replication-competent VSVΔG-LASV-GPC may provide an important advantage by eliciting these antibodies after a single vaccination.

Our data suggest that VSVΔG-LASV-GPC can elicit both antibodies that directly neutralize virus and that direct Fc-mediated effector functions, thus raising the possibility that both contribute to protection (Fig. 6). Interestingly, when we conducted a PCA to investigate which combinations of antibody characteristics were associated with protection, we found that the relative contribution of nAbs or Fc-directed effector functions appears to differ between individual animals (Fig. 6f–g). A protective role for non-neutralizing anti-GPC antibodies has been proposed previously after evaluating an inactivated whole virus vaccine based on a rabies virus recombinant that incorporated both rabies virus G and lineage IV LASV GPC in the viral envelope.^100^ Guinea pigs vaccinated three times with this vaccine were protected from challenge with a guinea pig-adapted LASV, and protection was associated with anti-GPC serum antibody titres, but not nAb activity. It is important to note that neutralizing and non-neutralizing functions are not mutually exclusive and likely work together to prevent progression of systemic infection and disease. Interestingly, non-neutralizing antibody effector functions targeting EBOV GP elicited by viral vectors including VSV also have been correlated with protection of NHPs.^46^^,^^64^

Activation of innate immune cells is essential to the induction of serum antibodies.^71^ Our analysis of the blood transcriptome demonstrated that VSVΔG-LASV-GPC induced an early innate anti-viral response detected 1 day following vaccination. A similar early innate response elicited by the VSVΔG-ZEBOV-GP vaccine is thought to play important roles in promoting protection including i) providing the adjuvant properties that drive development of protective adaptive immunity, and ii) inducing a fast-acting, transient antiviral effect that can delay disease progression while adaptive immunity develops when the vaccine is used to elicit rapid-onset protection.^28^^,^^35^^,^^36^^,^^72^ Notably, the rapid-onset component of protection was directly tested recently in a study conducted in macaques with an experimental VSVΔG-LASV-GPC vaccine. Results from this study demonstrated that vaccination as early as 3–7 days before LASV challenge protected the animals from lethal disease,^34^ like has been shown for a VSVΔG-based Ebola virus (Makona isolate) vaccine.^101^ Thus, we directly compared the early changes in the blood transcriptome after VSVΔG-LASV-GPC to those following VSVΔG-ZEBOV-GP vaccination^28^^,^^35^^,^^36^^,^^72^ and found substantial overlap, both in terms of individual DEGs and of transcriptome modules (Fig. 7e). Most of the shared DEGs encode proteins connected to the early antiviral state, including helicases (*DDX60* and *IFIH1*) involved in viral RNA sensing, proteins that antagonize viral replication (*OAS1*, *MX1*, *RSAD2*), and proteins that control the cellular response to IFN (*USP18*, *ISG15*, *HERC5*). Chemotactic factors also were modulated by both VSV-based vaccines (*CCL8* and *CCXCL10*). Interestingly, USP18 has been associated with allowing controlled replication of VSV and VSVΔG-ZEBOV-GP in infected macrophages as a mechanism to provide sufficient antigen to drive successful vaccination.^82^ Identifying DEGs that respond similarly to both the LASV and EBOV vaccines might provide a source of valuable biomarkers, which can be used during development of future VSVΔG-based vaccines to monitor vaccine performance in preclinical and clinical studies as well as provide a data bridge between vaccination responses induced in animals and people.

A primary limitation of this study was the comparatively small group sizes of 3–5 animals. These group sizes minimized use of NHPs while being sufficient to ensure evaluation of vaccine immunogenicity and efficacy and allow comparisons to earlier published preclinical data, but it did limit our analysis of potential sex-specific differences because the study was not powered to detect any subtle trends. Nonetheless, our data establish that VSVΔG-LASV-GPC effectively protected male and female animals from LASV infection.

In summary, we have shown that the VSVΔG-LASV-GPC preMVS created to support clinical development of the vaccine candidate was efficacious in the cynomolgus macaque model. A single dose protected macaques from lethal exposure to LASV 1 month after vaccination and this was associated with a diverse serum antibody response that included LASV lineage cross-neutralizing activity and Fc-mediated effector functions. Studies conducted in guinea pigs indicate that this immunity should be durable,^102^ and we currently are testing this in the macaque model by challenging animals 1 year after one or two vaccinations. We also showed that vaccination rapidly modulated expression of genes associated with the innate antiviral response, providing a gene expression signature that we are monitoring in human trials, including the multicentre phase 1 clinical trial that is completed [ClinicalTrials.gov NCT04794218 and^15^^,^^69^]. Induction of the early antiviral response might contribute to the rapid onset of protective immunity described for VSVΔG-LASV-GPC^34^ as well as other VSVΔG-based vaccines.^101^^,^^103^ The collective immune response data will provide a valuable point for comparison to ERVEBO and perhaps help build a profile of host responses to VSV-based vaccines that can be correlated with effective vaccination.

## Contributors

Conceptualization: CLC, GM, SBG, MBF, CLP.

Methodology: CLC, GM, MY, MLN, RWC, CW, KNA, VB, RPM, CA, VR, DG, FH, SL, LR, YC, AW, DW, OWS, AC, FG, DJF, ASE.

Investigation: CLC, GM, MY, MLN, RWC, CW, KNA, VB, RPM, CA, VR, DG, FH, SL, LR, YC, AW, DW, OWS, AC, FG, ASE, KMH, SRS, JWC, GA, EOS, JDA, TWG.

Visualization: CLC, GM, TSP, MLN, RPM, ASE.

Funding acquisition: ES, GA, EOS, JDA, TWG, SWG, MBF, CLP.

Project administration: DJF, AME, JDS.

Supervision: CLC, GM, RPM, KMH, GA, EOS, JDA, TWG, SBW, MBF, CLP.

Writing – original draft: CLC, GM, TSP, AK, CLP.

Writing – review & editing: CLC, GM, TSP, SRS, SBG, MBF, CLP.

All authors read and approved the final version of the manuscript. The underlying data was verified by TSP, SRS, and KMH.

## Data sharing statement

Code used for RNA-seq data analysis is available upon request. RNA-seq data used for transcriptomic analyses are available via Gene Expression Omnibus accession GSE246148.

The 3D map from the EM analysis has been deposited in the Electron Microscopy Databank (http://www.emdatabank.org/) under the following accession number EMD-42521. All data are available in the main text or the Supplementary materials.

## Declaration of interests

U.S. patent number 8,796,013 entitled “Pre- or Post-Exposure Treatment for Filovirus or Arenavirus Infection” issued to TWG on 5 August 2014 held by Boston University.

GA is a founder/equity holder in Seromyx Systems and Leyden Labs. GA has served as a scientific advisor for Sanofi Vaccines. GA has collaborative agreements with GSK, Merck, Abbvie, Sanofi, Medicago, BioNtech, Moderna, BMS, Novavax, SK Biosciences, Gilead, and Sanaria. RPM has collaborative agreements with Abbvie, Sanofi, Moderna, and Pfizer.

A U.S. provisional patent application (Serial No. 63/652,870) has been filed on the human vaccine candidate by IAVI (CLP, ES, MY, MBF).

## Appendix ASupplementary data

Supplementary Fig. S1 **Fig. S1. Detection of viral genome in blood of macaques after LASV challenge.** LASV genome copies in blood after challenge. Genome equivalents were quantified by RT-qPCR using an amplicon against the LASV NP gene. LOD, limit of detection. The study was terminated on day 28. Control animals were euthanized on days 11 and 13, as indicated. Data from viremic/RNAemic animal are indicated by their identifier (4 and 13). n = 3-5/group.

Supplementary Fig. S2 **Fig. S2. Individual components of the Clinical Score.** The Clinical Score shown in Fig. 3a incorporates data on (a) activity/appearance, (b) appetite, (c) bleeding/hemorrhage, (d) neurological signs, and (e) respiration. n = 3-5/group.

Supplementary Fig. S3 **Fig. S3. Profile of Ig isotypes and FcgR binding by anti-GPC serum antibodies.** Serum samples from days 0, 10 and 27 were analysed. (a–e) Titres of (a) IgG1, (b) IgG2, (c) IgG3, (d) IgA, and (e) IgM that bound to either the Lineage II or IV GP1-GP2 fusion protein (GP-link) or the prefusion GP. (f-j) FcgR-binding titre for antibodies that bound to Lineage II or IV GP1-GP2 fusion protein (GP-link) or the prefusion GP. Shown are log10-transformed fold changes relative to the baseline before vaccination. n = 3-5/group.

Supplementary Fig. S4 **Fig. S4. Association of immune responses with sex, dose and outcome.** (a) Sex is not a dominant variable for antibody responses induced by vaccination with VSVΔG-LASV-GPC. A PCA was built using all available data on antibody features. Colouring corresponds to sex of each animal. (b) Heatmap summarizing all data on adaptive immune responses 27 days after vaccination with VSVΔG-LASV-GPC. n = 3-5/group.

Supplementary Fig. S5 **Fig. S5. Effect of IM injection with VSVΔG-LASV-GPC on the whole-blood transcriptome.** Gene expression in whole blood samples from the high-dose (2x10ˆ7 PFU), low-dose (2x10ˆ5 PFU) and control groups were analysed by RNA-seq prior to and on days 1 and 3 after vaccination. Heatmap showing normalized expression values of all expressed genes with unsupervised hierarchical clustering. n = 3-5/group.

Supplementary Fig. S6 **Fig. S6. Known interactions between the genes identified as differentially expressed genes and sex bias.** STRING interactome for DEGs identified after VSVΔG-LASV-GPC vaccination.

Supplementary Fig. S7 **Fig. S7. Supplemental analyses of genes exhibiting differential expression after vaccination with VSVΔG-LASV-GPC.** (a) Venn diagram showing the overlap in gene sets enriched for DEGs after vaccination with VSVΔG-LASV-GPC and VSVΔG-ZEBOV-GP. “DC”, “LI” and “HM” prefixes on gene sets indicate the published collection from which they originated, respectively. (b) Overlap of vaccination-induced DEGs with genes differentially expressed between male (M) and female (F) animals in each group. DEGs from days 1 and 3 after vaccination were combined into a single list for this analysis.

Supplementary Table S4

Cooper et al_VSV LASV_ARRIVE

## References

[1] Garry R.F. (2023). Lassa fever - the road ahead. Nat Rev Microbiol.

[2] Basinski A.J., Fichet-Calvet E., Sjodin A.R. (2021). Bridging the gap: using reservoir ecology and human serosurveys to estimate Lassa virus spillover in West Africa. PLoS Comput Biol.

[3] Klitting R., Kafetzopoulou L.E., Thiery W. (2022). Predicting the evolution of the Lassa virus endemic area and population at risk over the next decades. Nat Commun.

[4] Raabe V., Mehta A.K., Evans J.D., Science Working Group of the National Emerging Special Pathogens T, Education Center Special Pathogens Research N (2022). Lassa virus infection: a summary for clinicians. Int J Infect Dis.

[5] Mehand M.S., Al-Shorbaji F., Millett P., Murgue B. (2018). The WHO R&D Blueprint: 2018 review of emerging infectious diseases requiring urgent research and development efforts. Antiviral Res.

[6] Prescott J.B., Marzi A., Safronetz D., Robertson S.J., Feldmann H., Best S.M. (2017). Immunobiology of Ebola and lassa virus infections. Nat Rev Immunol.

[7] Murphy H., Ly H. (2022). Understanding immune responses to lassa virus infection and to its candidate vaccines. Vaccines (Basel).

[8] Baillet N., Reynard S., Perthame E. (2021). Systemic viral spreading and defective host responses are associated with fatal Lassa fever in macaques. Commun Biol.

[9] Baize S., Marianneau P., Loth P. (2009). Early and strong immune responses are associated with control of viral replication and recovery in lassa virus-infected cynomolgus monkeys. J Virol.

[10] Cross R.W., Heinrich M.L., Fenton K.A. (2023). A human monoclonal antibody combination rescues nonhuman primates from advanced disease caused by the major lineages of Lassa virus. Proc Natl Acad Sci USA.

[11] Mire C.E., Cross R.W., Geisbert J.B. (2017). Human-monoclonal-antibody therapy protects nonhuman primates against advanced Lassa fever. Nat Med.

[12] Cross R.W., Fenton K.A., Woolsey C. (2024). Monoclonal antibody therapy protects nonhuman primates against mucosal exposure to Lassa virus. Cell Rep Med.

[13] Salami K., Gouglas D., Schmaljohn C., Saville M., Tornieporth N. (2019). A review of Lassa fever vaccine candidates. Curr Opin Virol.

[14] Saito T., Reyna R.A., Taniguchi S., Littlefield K., Paessler S., Maruyama J. (2023). Vaccine candidates against arenavirus infections. Vaccines (Basel).

[15] Sulis G., Peebles A., Basta N.E. (2023). Lassa fever vaccine candidates: a scoping review of vaccine clinical trials. Trop Med Int Health.

[16] McCormick J.B., Mitchell S.W., Kiley M.P., Ruo S., Fisher-Hoch S.P. (1992). Inactivated Lassa virus elicits a non protective immune response in rhesus monkeys. J Med Virol.

[17] Hastie K.M., Zandonatti M.A., Kleinfelter L.M. (2017). Structural basis for antibody-mediated neutralization of Lassa virus. Science.

[18] Gorman J., Cheung C.S., Duan Z. (2024). Cleavage-intermediate Lassa virus trimer elicits neutralizing responses, identifies neutralizing nanobodies, and reveals an apex-situated site-of-vulnerability. Nat Commun.

[19] Perrett H.R., Brouwer P.J.M., Hurtado J. (2023). Structural conservation of Lassa virus glycoproteins and recognition by neutralizing antibodies. Cell Rep.

[20] Katz M., Weinstein J., Eilon-Ashkenazy M. (2022). Structure and receptor recognition by the Lassa virus spike complex. Nature.

[21] Amanna I.J., Slifka M.K. (2020). Successful vaccines. Curr Top Microbiol Immunol.

[22] Biedenkopf N., Bukreyev A., Chandran K. (2023). Renaming of genera Ebolavirus and Marburgvirus to Orthoebolavirus and Orthomarburgvirus, respectively, and introduction of binomial species names within family Filoviridae. Arch Virol.

[23] Jones S.M., Feldmann H., Stroher U. (2005). Live attenuated recombinant vaccine protects nonhuman primates against Ebola and Marburg viruses. Nat Med.

[24] Wolf J., Jannat R., Dubey S. (2021). Development of pandemic vaccines: ERVEBO case study. Vaccines (Basel).

[25] Garbutt M., Liebscher R., Wahl-Jensen V. (2004). Properties of replication-competent vesicular stomatitis virus vectors expressing glycoproteins of filoviruses and arenaviruses. J Virol.

[26] Mire C.E., Miller A.D., Carville A. (2012). Recombinant vesicular stomatitis virus vaccine vectors expressing filovirus glycoproteins lack neurovirulence in nonhuman primates. PLoS Negl Trop Dis.

[27] Monath T.P., Fast P.E., Modjarrad K. (2019). rVSVDeltaG-ZEBOV-GP (also designated V920) recombinant vesicular stomatitis virus pseudotyped with Ebola Zaire Glycoprotein: standardized template with key considerations for a risk/benefit assessment. Vaccine X.

[28] Pinski A.N., Messaoudi I. (2020). To B or not to B: mechanisms of protection conferred by rVSV-EBOV-GP and the roles of innate and adaptive immunity. Microorganisms.

[29] Henao-Restrepo A.M., Camacho A., Longini I.M. (2017). Efficacy and effectiveness of an rVSV-vectored vaccine in preventing Ebola virus disease: final results from the Guinea ring vaccination, open-label, cluster-randomised trial (Ebola Ca Suffit!). Lancet.

[30] Grais R.F., Kennedy S.B., Mahon B.E. (2021). Estimation of the correlates of protection of the rVSVDeltaG-ZEBOV-GP Zaire ebolavirus vaccine: a post-hoc analysis of data from phase 2/3 clinical trials. Lancet Microbe.

[31] Safronetz D., Mire C., Rosenke K. (2015). A recombinant vesicular stomatitis virus-based Lassa fever vaccine protects Guinea pigs and macaques against challenge with geographically and genetically distinct Lassa viruses. PLoS Negl Trop Dis.

[32] Marzi A., Feldmann F., Geisbert T.W., Feldmann H., Safronetz D. (2015). Vesicular stomatitis virus-based vaccines against Lassa and Ebola viruses. Emerg Infect Dis.

[33] Geisbert T.W., Jones S., Fritz E.A. (2005). Development of a new vaccine for the prevention of Lassa fever. PLoS medicine.

[34] Cross R.W., Woolsey C., Prasad A.N. (2022). A recombinant VSV-vectored vaccine rapidly protects nonhuman primates against heterologous lethal Lassa fever. Cell Rep.

[35] Santoro F., Donato A., Lucchesi S. (2021). Human transcriptomic response to the VSV-vectored Ebola vaccine. Vaccines (Basel).

[36] Vianello E., Gonzalez-Dias P., van Veen S. (2022). Transcriptomic signatures induced by the Ebola virus vaccine rVSVDeltaG-ZEBOV-GP in adult cohorts in Europe, Africa, and North America: a molecular biomarker study. Lancet Microbe.

[37] Espeseth A.S., Yuan M., Citron M. (2022). Preclinical immunogenicity and efficacy of a candidate COVID-19 vaccine based on a vesicular stomatitis virus-SARS-CoV-2 chimera. EBioMedicine.

[38] Cooper C.L., Morrow G., Yuan M. (2022). Nonhuman primates are protected against Marburg virus disease by vaccination with a vesicular stomatitis virus vector-based vaccine prepared under conditions to allow advancement to human clinical trials. Vaccines (Basel).

[39] Rabinovich S., Powell R.L., Lindsay R.W. (2014). A novel, live-attenuated vesicular stomatitis virus vector displaying conformationally intact, functional HIV-1 envelope trimers that elicits potent cellular and humoral responses in mice. PLoS One.

[40] Robinson J.E., Hastie K.M., Cross R.W. (2016). Most neutralizing human monoclonal antibodies target novel epitopes requiring both Lassa virus glycoprotein subunits. Nat Commun.

[41] Walker L.M., Huber M., Doores K.J. (2011). Broad neutralization coverage of HIV by multiple highly potent antibodies. Nature.

[42] Lyles D.S., Puddington L., McCreedy B.J. (1988). Vesicular stomatitis virus M protein in the nuclei of infected cells. J Virol.

[43] Punjani A., Rubinstein J.L., Fleet D.J., Brubaker M.A. (2017). cryoSPARC: algorithms for rapid unsupervised cryo-EM structure determination. Nat Methods.

[44] Pettersen E.F., Goddard T.D., Huang C.C. (2021). UCSF ChimeraX: structure visualization for researchers, educators, and developers. Protein Sci.

[45] Gill D.K., Huang Y., Levine G.L. (2010). Equivalence of ELISpot assays demonstrated between major HIV network laboratories. PLoS One.

[46] Meyer M., Gunn B.M., Malherbe D.C. (2021). Ebola vaccine-induced protection in nonhuman primates correlates with antibody specificity and Fc-mediated effects. Sci Transl Med.

[47] Bartsch Y.C., Wang C., Zohar T. (2021). Humoral signatures of protective and pathological SARS-CoV-2 infection in children. Nat Med.

[48] Brown E.P., Dowell K.G., Boesch A.W. (2017). Multiplexed Fc array for evaluation of antigen-specific antibody effector profiles. J Immunol Methods.

[49] Dobin A., Davis C.A., Schlesinger F. (2013). STAR: ultrafast universal RNA-seq aligner. Bioinformatics.

[50] Liao Y., Smyth G.K., Shi W. (2014). featureCounts: an efficient general purpose program for assigning sequence reads to genomic features. Bioinformatics.

[51] Love M.I., Huber W., Anders S. (2014). Moderated estimation of fold change and dispersion for RNA-seq data with DESeq2. Genome Biol.

[52] Weiner J., Domaszewska T. (2016). tmod: an R package for general and multivariate enrichment analysis. PeerJ Preprints.

[53] Liberzon A., Birger C., Thorvaldsdottir H., Ghandi M., Mesirov J.P., Tamayo P. (2015). The Molecular Signatures Database (MSigDB) hallmark gene set collection. Cell Syst.

[54] Heinz S., Benner C., Spann N. (2010). Simple combinations of lineage-determining transcription factors prime cis-regulatory elements required for macrophage and B cell identities. Mol Cell.

[55] Szklarczyk D., Gable A.L., Lyon D. (2019). STRING v11: protein-protein association networks with increased coverage, supporting functional discovery in genome-wide experimental datasets. Nucleic Acids Res.

[56] Dunn O.J. (1964). Multiple comparisons using rank sums. Technometrics.

[57] Benjamini Y., Hochberg Y. (1995). Controlling the false discovery rate: a practical and powerful approach to multiple testing. J R Stat Soc B (Methodological).

[58] Barr J.N., Whelan S.P., Wertz G.W. (2002). Transcriptional control of the RNA-dependent RNA polymerase of vesicular stomatitis virus. Biochim Biophys Acta.

[59] Prout A., Rustandi R.R., Tubbs C., Winters M.A., McKenna P., Vlasak J. (2022). Functional profiling of Covid 19 vaccine candidate by flow virometry. Vaccine.

[60] Hensley L.E., Smith M.A., Geisbert J.B. (2011). Pathogenesis of Lassa fever in cynomolgus macaques. Virol J.

[61] Sattler R.A., Paessler S., Ly H., Huang C. (2020). Animal models of lassa fever. Pathogens.

[62] Enriquez A.S., Buck T.K., Li H. (2022). Delineating the mechanism of anti-Lassa virus GPC-A neutralizing antibodies. Cell Rep.

[63] Bachmann M.F., Jennings G.T. (2010). Vaccine delivery: a matter of size, geometry, kinetics and molecular patterns. Nat Rev Immunol.

[64] Gunn B.M., McNamara R.P., Wood L. (2023). Antibodies against the Ebola virus soluble glycoprotein are associated with long-term vaccine-mediated protection of non-human primates. Cell Rep.

[65] McNamara R.P., Maron J.S., Boucau J. (2023). Anamnestic humoral correlates of immunity across SARS-CoV-2 variants of concern. mBio.

[66] Gunn B.M., Bai S. (2021). Building a better antibody through the Fc: advances and challenges in harnessing antibody Fc effector functions for antiviral protection. Hum Vaccines Immunother.

[67] Tong X., McNamara R.P., Avendano M.J. (2023). Waning and boosting of antibody Fc-effector functions upon SARS-CoV-2 vaccination. Nat Commun.

[68] Danesh A., Ren Y., Brad Jones R. (2020). Roles of fragment crystallizable-mediated effector functions in broadly neutralizing antibody activity against HIV. Curr Opin HIV AIDS.

[69] Moore K.A., Ostrowsky J.T., Mehr A.J. (2024). Lassa fever research priorities: towards effective medical countermeasures by the end of the decade. Lancet Infect Dis.

[70] Kaplonek P., Deng Y., Shih-Lu Lee J. (2023). Hybrid immunity expands the functional humoral footprint of both mRNA and vector-based SARS-CoV-2 vaccines. Cell Rep Med.

[71] Iwasaki A., Medzhitov R. (2015). Control of adaptive immunity by the innate immune system. Nat Immunol.

[72] Rechtien A., Richert L., Lorenzo H. (2017). Systems vaccinology identifies an early innate immune signature as a correlate of antibody responses to the Ebola vaccine rVSV-ZEBOV. Cell Rep.

[73] Li S., Rouphael N., Duraisingham S. (2014). Molecular signatures of antibody responses derived from a systems biology study of five human vaccines. Nat Immunol.

[74] Chaussabel D., Quinn C., Shen J. (2008). A modular analysis framework for blood genomics studies: application to systemic lupus erythematosus. Immunity.

[75] Cross R.W., Xu R., Matassov D. (2020). Quadrivalent VesiculoVax vaccine protects nonhuman primates from viral-induced hemorrhagic fever and death. J Clin Invest.

[76] Masopust D., Vezys V., Marzo A.L., Lefrancois L. (2001). Preferential localization of effector memory cells in nonlymphoid tissue. Science.

[77] Hangartner L., Zinkernagel R.M., Hengartner H. (2006). Antiviral antibody responses: the two extremes of a wide spectrum. Nat Rev Immunol.

[78] Pannetier D., Reynard S., Russier M. (2011). Human dendritic cells infected with the nonpathogenic Mopeia virus induce stronger T-cell responses than those infected with Lassa virus. J Virol.

[79] Baize S., Kaplon J., Faure C., Pannetier D., Georges-Courbot M.C., Deubel V. (2004). Lassa virus infection of human dendritic cells and macrophages is productive but fails to activate cells. J Immunol.

[80] Mahanty S., Hutchinson K., Agarwal S., McRae M., Rollin P.E., Pulendran B. (2003). Cutting edge: impairment of dendritic cells and adaptive immunity by Ebola and Lassa viruses. J Immunol.

[81] Schaeffer J., Carnec X., Reynard S. (2018). Lassa virus activates myeloid dendritic cells but suppresses their ability to stimulate T cells. PLoS Pathog.

[82] Friedrich S.K., Schmitz R., Bergerhausen M. (2020). Usp18 expression in CD169(+) macrophages is important for strong immune response after vaccination with VSV-EBOV. Vaccines (Basel).

[83] Tomczyk T., Wrobel G., Chaber R. (2018). Immune consequences of in vitro infection of human peripheral blood leukocytes with vesicular stomatitis virus. J Innate Immun.

[84] Boudreau J.E., Bridle B.W., Stephenson K.B. (2009). Recombinant vesicular stomatitis virus transduction of dendritic cells enhances their ability to prime innate and adaptive antitumor immunity. Mol Ther.

[85] Howerth E.W., Mead D.G., Mueller P.O., Duncan L., Murphy M.D., Stallknecht D.E. (2006). Experimental vesicular stomatitis virus infection in horses: effect of route of inoculation and virus serotype. Vet Pathol.

[86] Raabe V., Lai L., Morales J. (2023). Cellular and humoral immunity to Ebola Zaire glycoprotein and viral vector proteins following immunization with recombinant vesicular stomatitis virus-based Ebola vaccine (rVSVDeltaG-ZEBOV-GP). Vaccine.

[87] Poetsch J.H., Dahlke C., Zinser M.E. (2019). Detectable vesicular stomatitis virus (VSV)-Specific humoral and cellular immune responses following VSV-Ebola virus vaccination in humans. J Infect Dis.

[88] Wiedemann A., Lhomme E., Huchon M. (2024). Long-term cellular immunity of vaccines for Zaire Ebola virus diseases. Nat Commun.

[89] Simon J.K., Kennedy S.B., Mahon B.E. (2022). Immunogenicity of rVSVDeltaG-ZEBOV-GP Ebola vaccine (ERVEBO(R)) in African clinical trial participants by age, sex, and baseline GP-ELISA titer: a post hoc analysis of three Phase 2/3 trials. Vaccine.

[90] Ehrhardt S.A., Zehner M., Krahling V. (2019). Polyclonal and convergent antibody response to Ebola virus vaccine rVSV-ZEBOV. Nat Med.

[91] Wilkinson D.E., Page M., Mattiuzzo G. (2017). Comparison of platform technologies for assaying antibody to Ebola virus. Vaccine.

[92] Heppner D.G., Kemp T.L., Martin B.K. (2017). Safety and immunogenicity of the rVSVΔG-ZEBOV-GP Ebola virus vaccine candidate in healthy adults: a phase 1b randomised, multicentre, double-blind, placebo-controlled, dose-response study. Lancet Infect Dis.

[93] Halperin S.A., Das R., Onorato M.T. (2019). Immunogenicity, Lot consistency, and extended safety of rVSVDeltaG-ZEBOV-GP vaccine: a phase 3 randomized, double-blind, placebo-controlled study in healthy adults. J Infect Dis.

[94] Agnandji S.T., Huttner A., Zinser M.E. (2016). Phase 1 trials of rVSV Ebola vaccine in Africa and Europe. New England J Med.

[95] Jahrling P.B., Peters C.J. (1984). Passive antibody therapy of Lassa fever in cynomolgus monkeys: importance of neutralizing antibody and Lassa virus strain. Infect Immun.

[96] Cross R.W., Mire C.E., Branco L.M. (2016). Treatment of Lassa virus infection in outbred Guinea pigs with first-in-class human monoclonal antibodies. Antiviral Res.

[97] Brouwer P.J.M., Antanasijevic A., Ronk A.J. (2022). Lassa virus glycoprotein nanoparticles elicit neutralizing antibody responses and protection. Cell Host Microbe.

[98] Mateo M., Reynard S., Journeaux A. (2021). A single-shot Lassa vaccine induces long-term immunity and protects cynomolgus monkeys against heterologous strains. Sci Transl Med.

[99] Muller H., Fehling S.K., Dorna J. (2020). Adjuvant formulated virus-like particles expressing native-like forms of the Lassa virus envelope surface glycoprotein are immunogenic and induce antibodies with broadly neutralizing activity. NPJ Vaccines.

[100] Abreu-Mota T., Hagen K.R., Cooper K. (2018). Non-neutralizing antibodies elicited by recombinant Lassa-Rabies vaccine are critical for protection against Lassa fever. Nat Commun.

[101] Marzi A., Robertson S.J., Haddock E. (2015). EBOLA VACCINE. VSV-EBOV rapidly protects macaques against infection with the 2014/15 Ebola virus outbreak strain. Science.

[102] Stein D.R., Warner B.M., Soule G. (2019). A recombinant vesicular stomatitis-based Lassa fever vaccine elicits rapid and long-term protection from lethal Lassa virus infection in Guinea pigs. NPJ Vaccines.

[103] Marzi A., Jankeel A., Menicucci A.R. (2021). Single dose of a VSV-based vaccine rapidly protects macaques from Marburg virus disease. Front Immunol.

