# Supplementary figures and images for: Preclinical development of a replication-competent vesicular stomatitis virus-based Lassa virus vaccine candidate advanced into human clinical trials
Source: eBioMedicine. 2025 Mar 28;114:105647. doi: 10.1016/j.ebiom.2025.105647 (PMC11994357; doi:10.1016/j.ebiom.2025.105647)

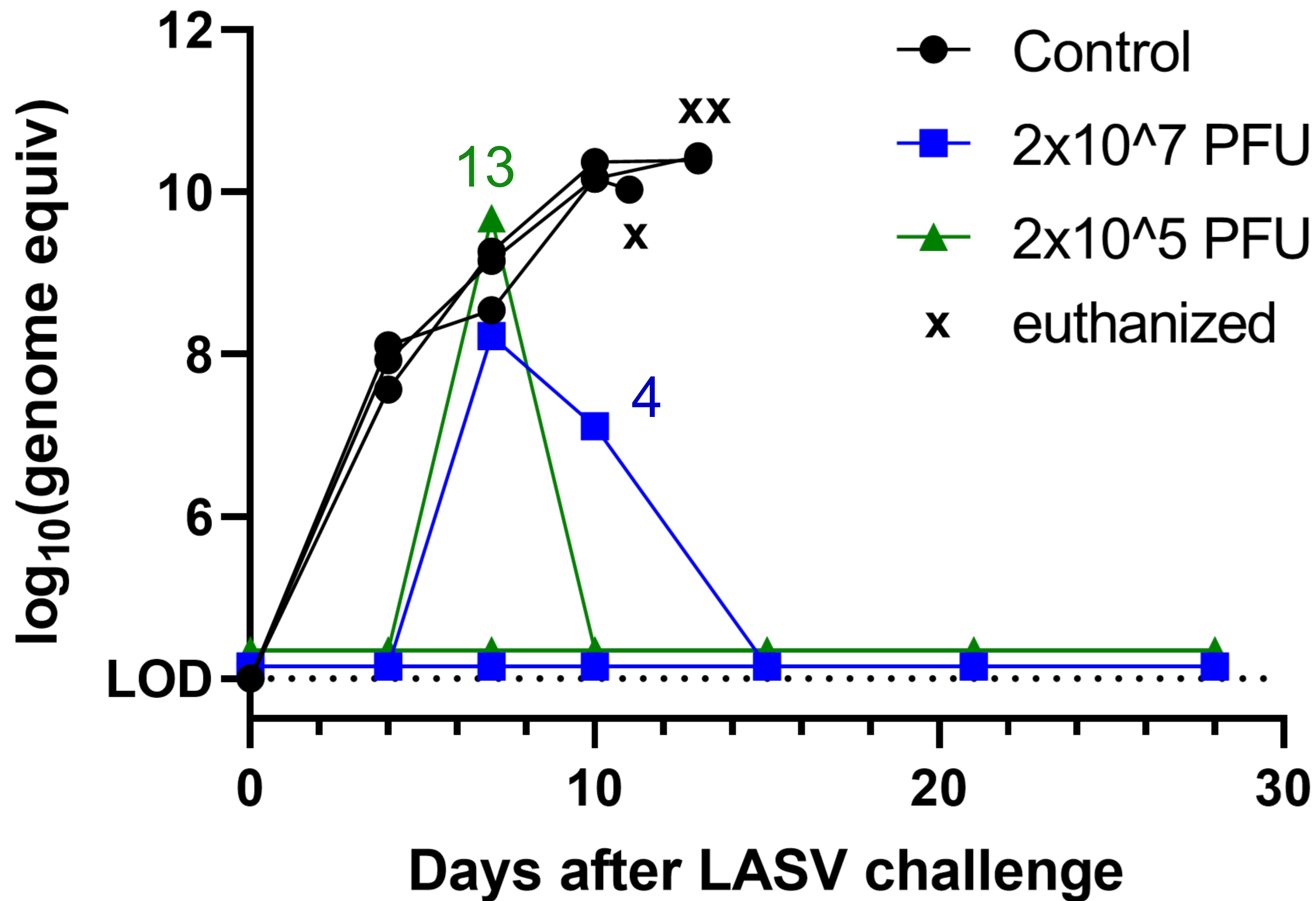

Supplement: Supplementary Fig. S1 — Fig. S1. Detection of viral genome in blood of macaques after LASV challenge. LASV genome copies in blood after challenge. Genome equivalents were quantified by RT-qPCR using an amplicon against the LASV NP gene. LOD, limit of detection. The study was terminated on day 28. Control animals were euthanized on days 11 and 13, as indicated. Data from viremic/RNAemic animal are indicated by their identifier (4 and 13). n = 3-5/group. [file mmc1.pdf]

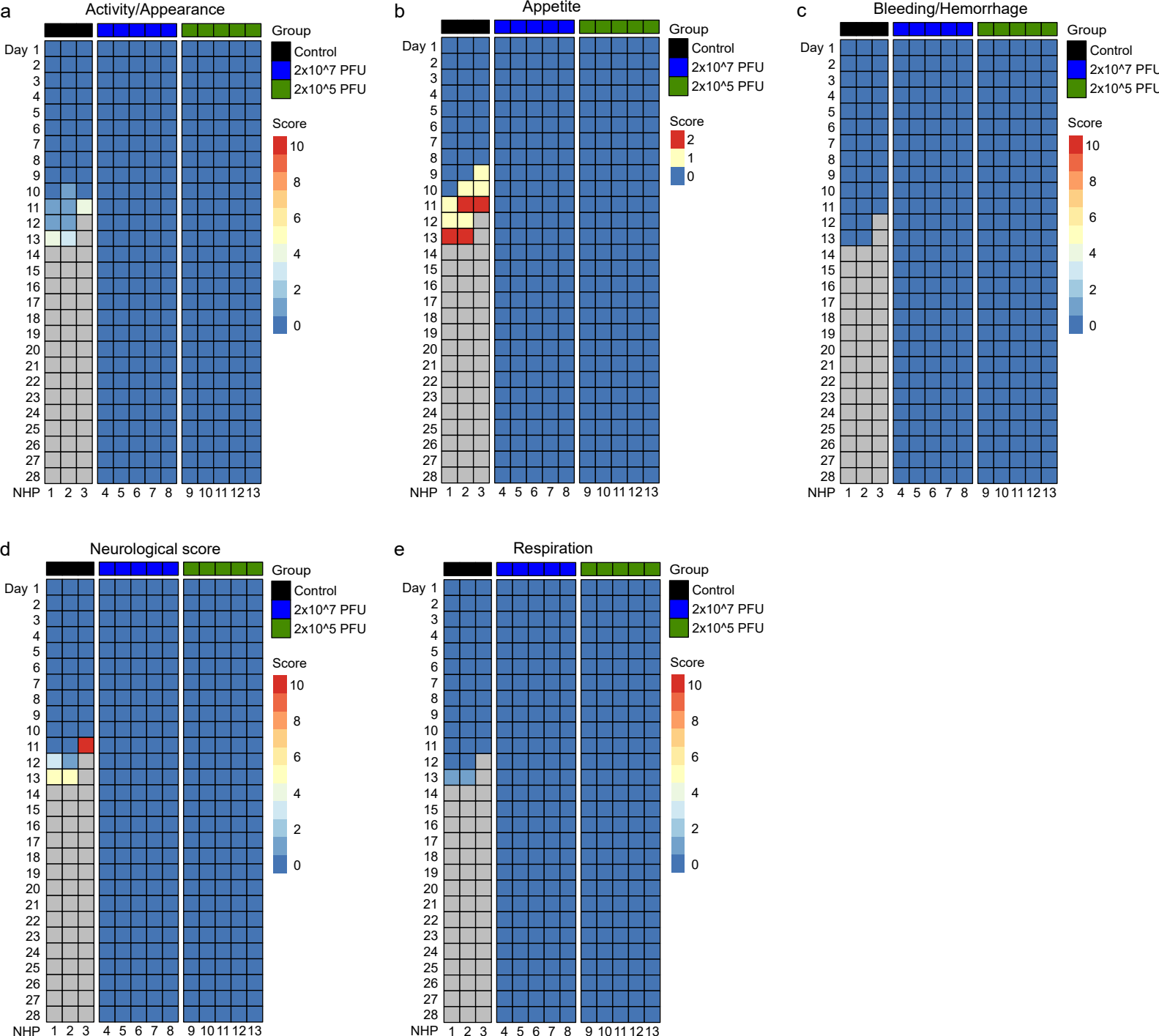

Supplement: Supplementary Fig. S2 — Fig. S2. Individual components of the Clinical Score. The Clinical Score shown in Fig. 3a incorporates data on (a) activity/appearance, (b) appetite, (c) bleeding/hemorrhage, (d) neurological signs, and (e) respiration. n = 3-5/group. [file mmc2.pdf]

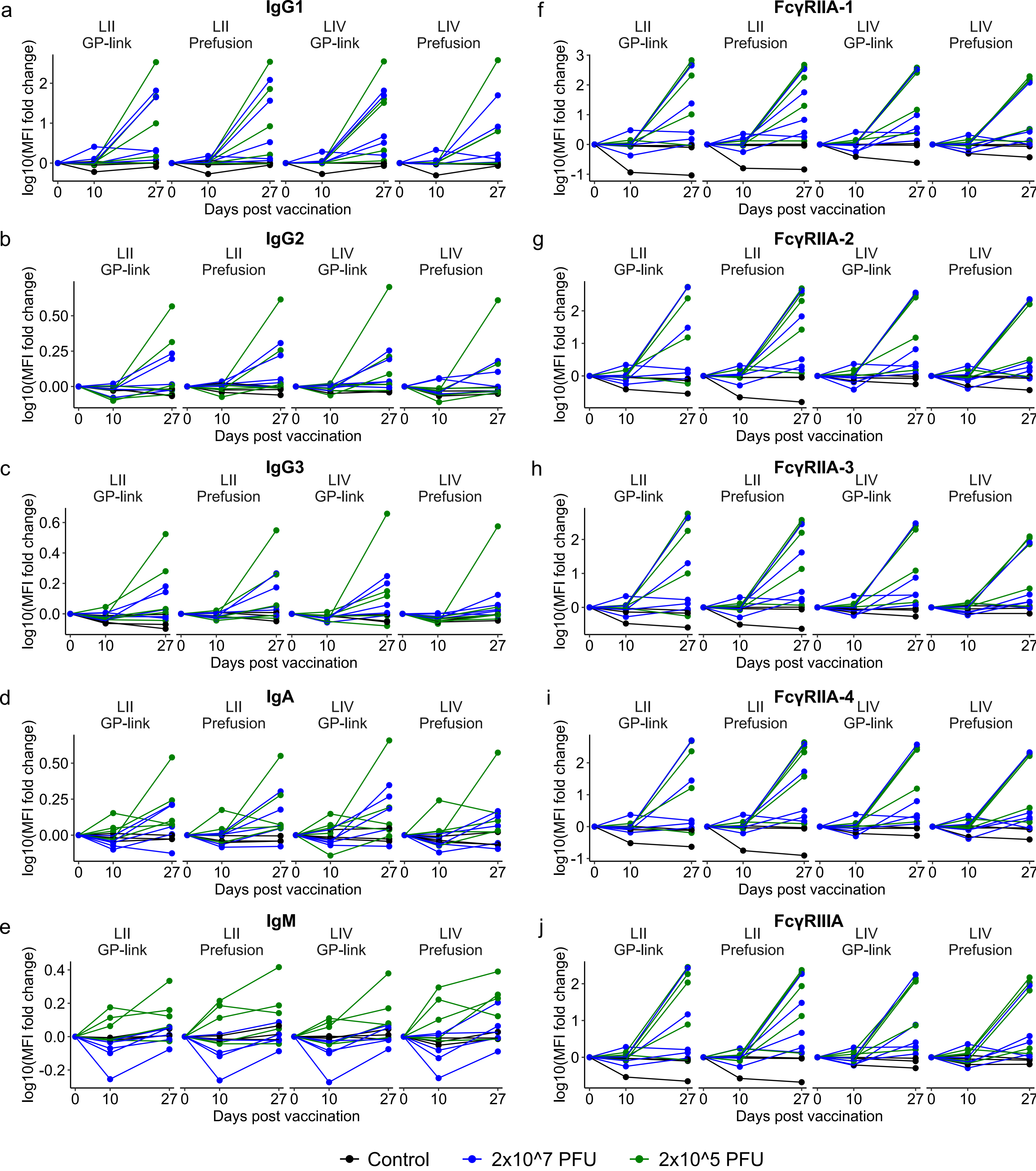

Supplement: Supplementary Fig. S3 — Fig. S3. Profile of Ig isotypes and FcgR binding by anti-GPC serum antibodies. Serum samples from days 0, 10 and 27 were analysed. (a–e) Titres of (a) IgG1, (b) IgG2, (c) IgG3, (d) IgA, and (e) IgM that bound to either the Lineage II or IV GP1-GP2 fusion protein (GP-link) or the prefusion GP. (f-j) FcgR-binding titre for antibodies that bound to Lineage II or IV GP1-GP2 fusion protein (GP-link) or the prefusion GP. Shown are log10-transformed fold changes relative to the baseline before vaccination. n = 3-5/group. [file mmc3.pdf]

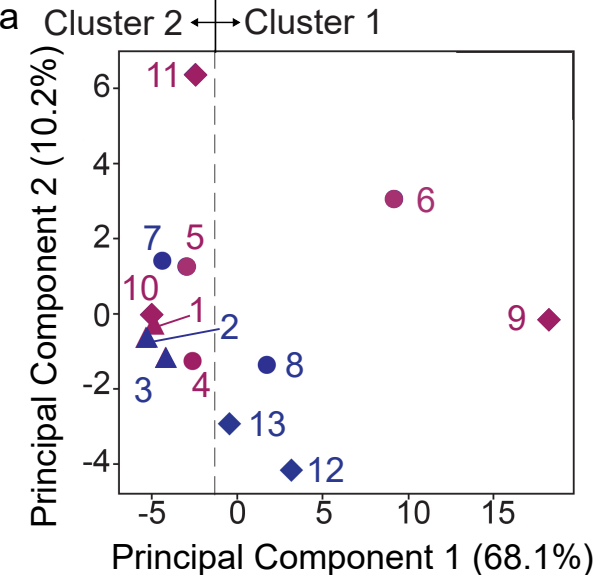

**Sex**

■ Male

■ Female

**Dose**

▲ Control

●  $2 \times 10^7$  PFU

◆  $2 \times 10^5$  PFU

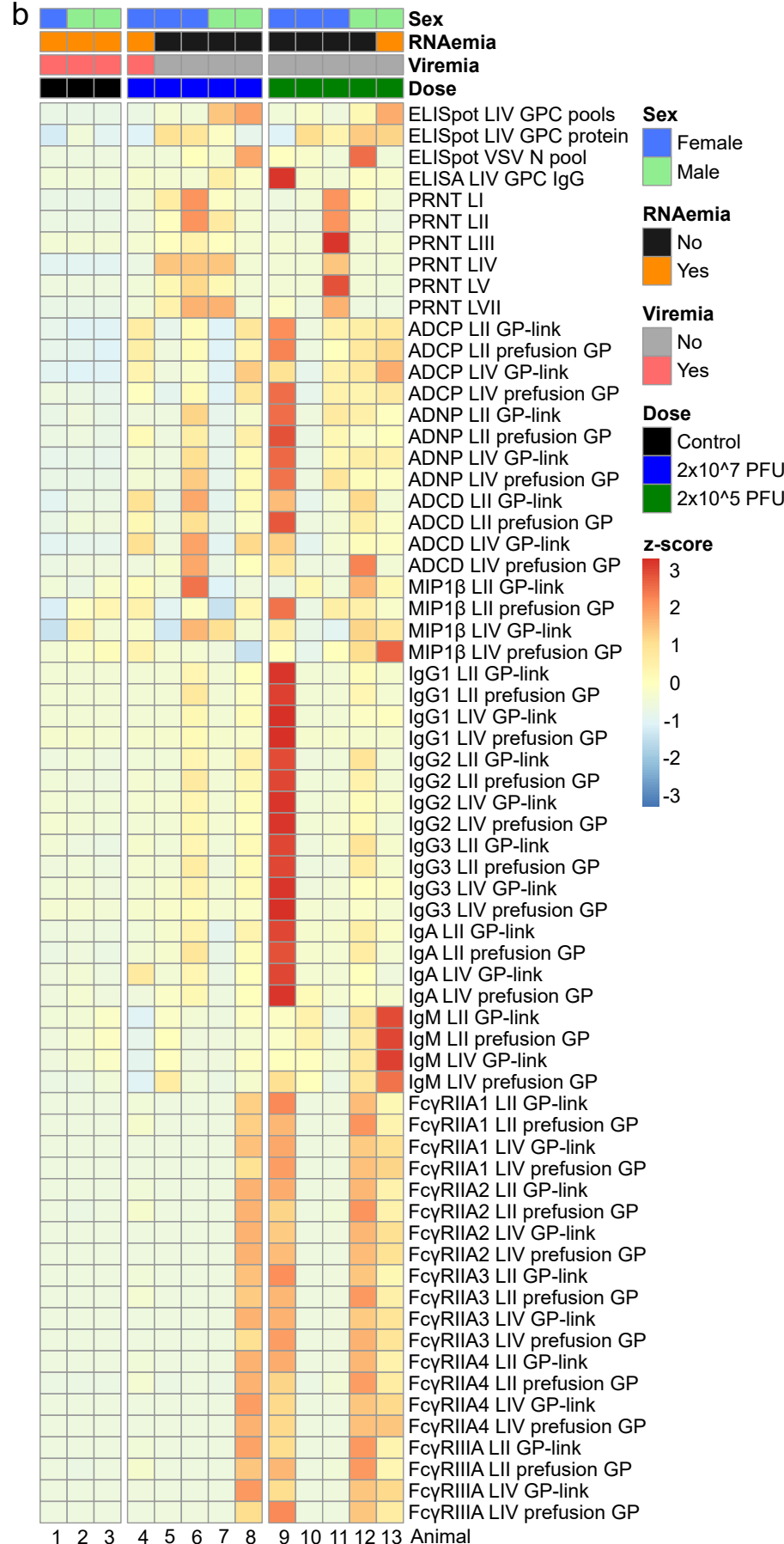

Supplement: Supplementary Fig. S4 — Fig. S4. Association of immune responses with sex, dose and outcome. (a) Sex is not a dominant variable for antibody responses induced by vaccination with VSVΔG-LASV-GPC. A PCA was built using all available data on antibody features. Colouring corresponds to sex of each animal. (b) Heatmap summarizing all data on adaptive immune responses 27 days after vaccination with VSVΔG-LASV-GPC. n = 3-5/group. [file mmc4.pdf]

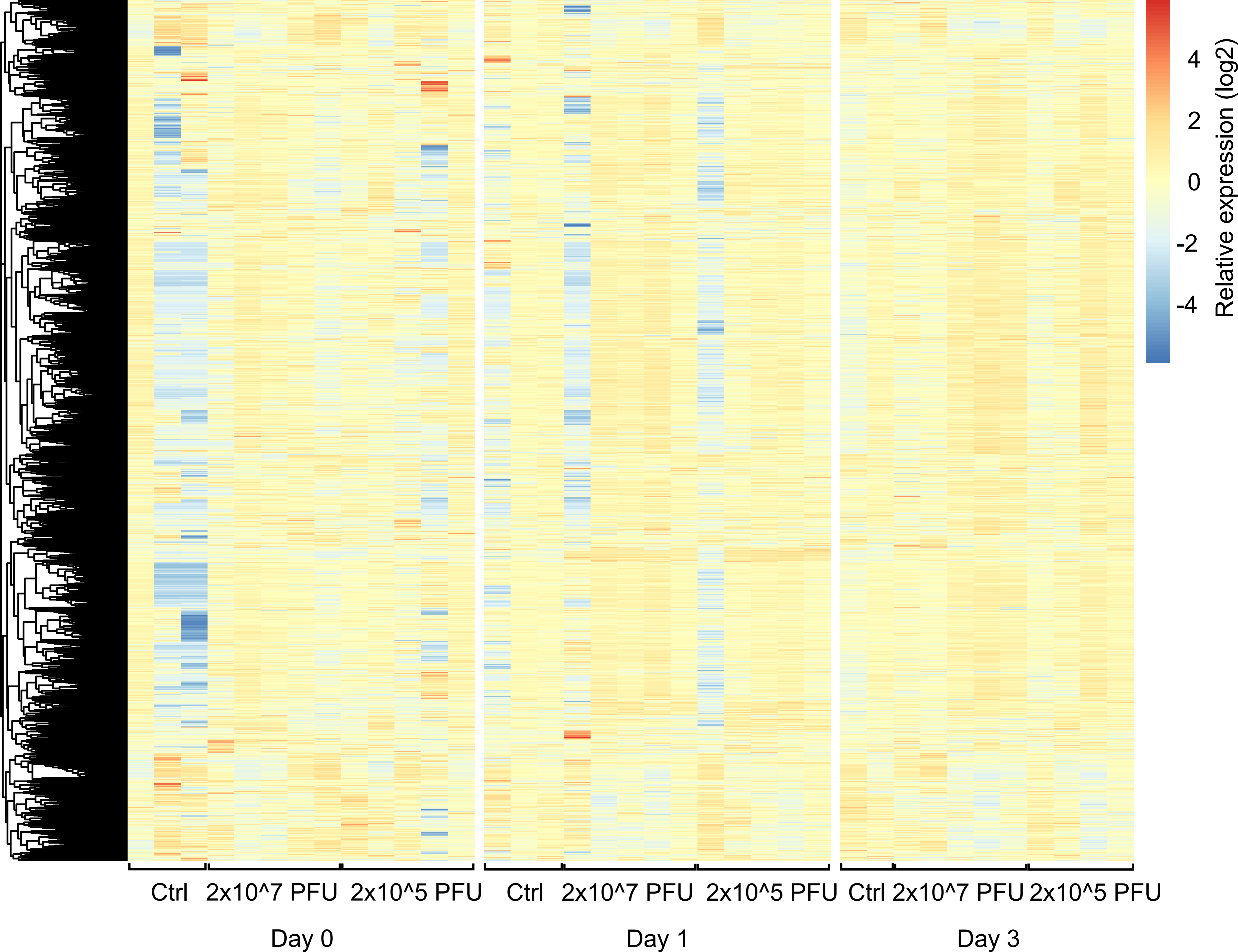

Supplement: Supplementary Fig. S5 — Fig. S5. Effect of IM injection with VSVΔG-LASV-GPC on the whole-blood transcriptome. Gene expression in whole blood samples from the high-dose (2x10ˆ7 PFU), low-dose (2x10ˆ5 PFU) and control groups were analysed by RNA-seq prior to and on days 1 and 3 after vaccination. Heatmap showing normalized expression values of all expressed genes with unsupervised hierarchical clustering. n = 3-5/group. [file mmc5.pdf]

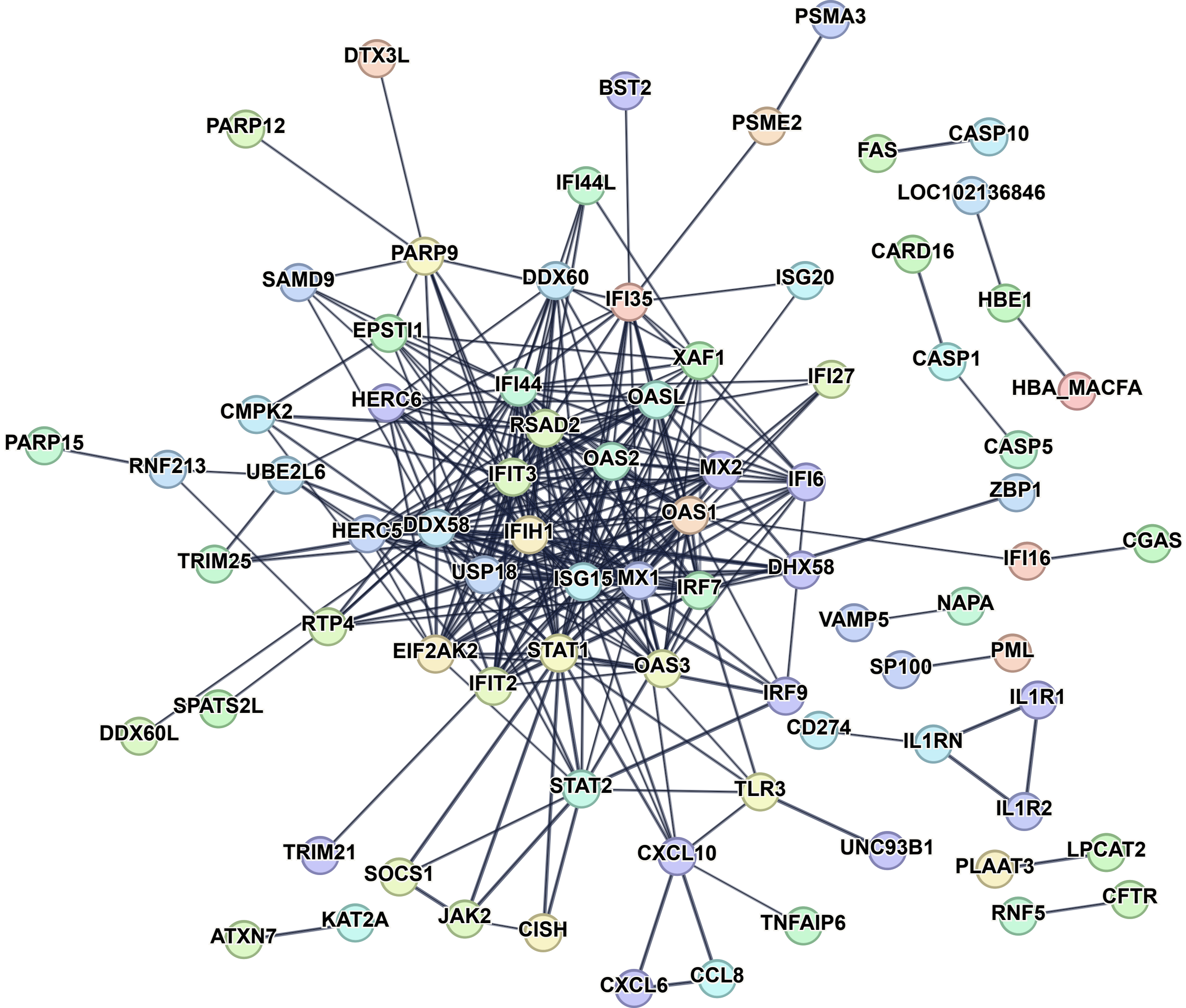

Supplement: Supplementary Fig. S6 — Fig. S6. Known interactions between the genes identified as differentially expressed genes and sex bias. STRING interactome for DEGs identified after VSVΔG-LASV-GPC vaccination. [file mmc6.pdf]

a

VSVΔG-ZEBOV-GP

VSVΔG-LASV-GPC

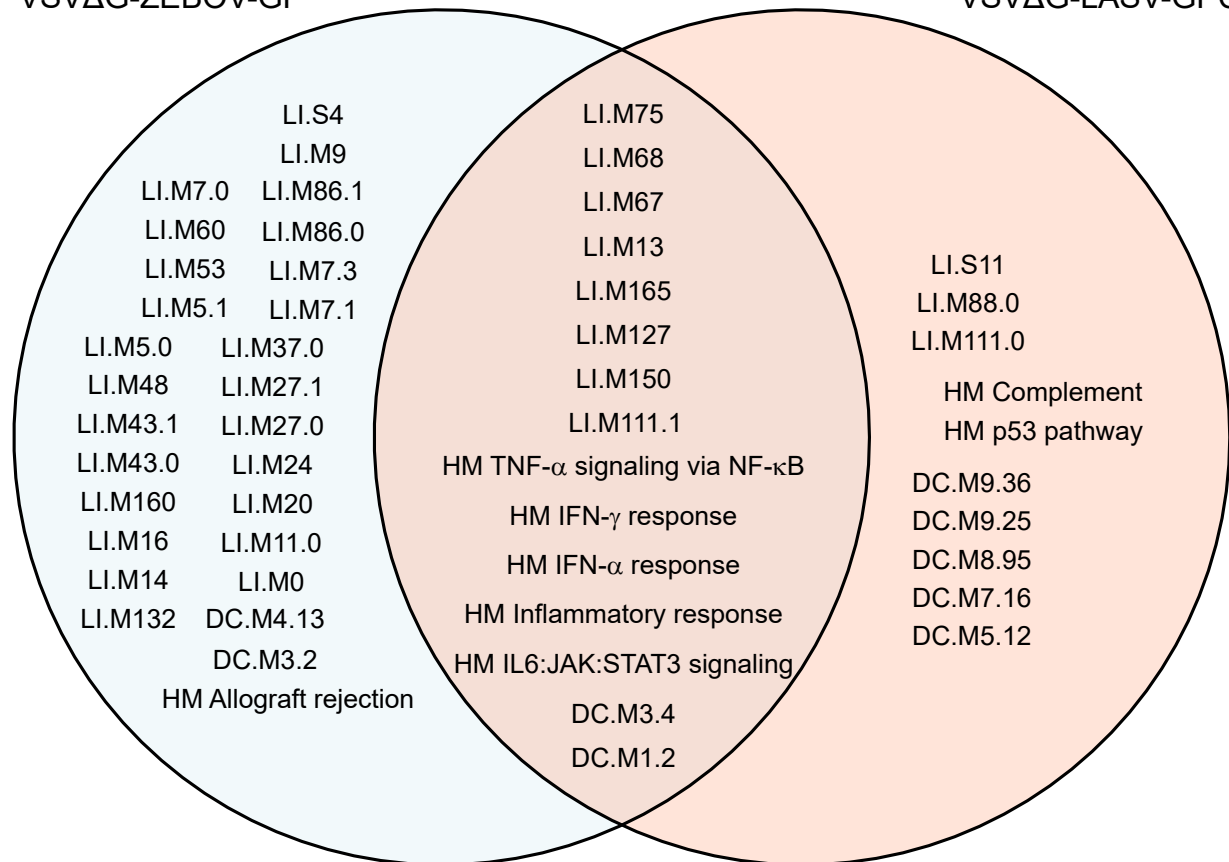

b

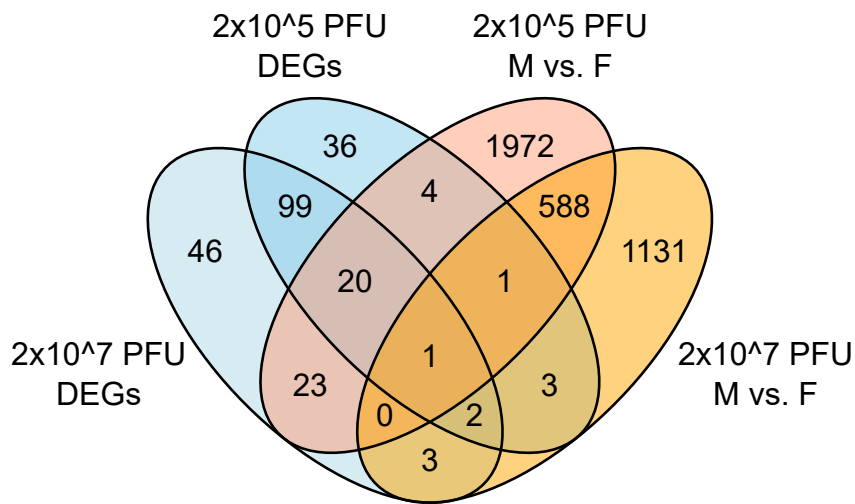

Supplement: Supplementary Fig. S7 — Fig. S7. Supplemental analyses of genes exhibiting differential expression after vaccination with VSVΔG-LASV-GPC. (a) Venn diagram showing the overlap in gene sets enriched for DEGs after vaccination with VSVΔG-LASV-GPC and VSVΔG-ZEBOV-GP. “DC”, “LI” and “HM” prefixes on gene sets indicate the published collection from which they originated, respectively. (b) Overlap of vaccination-induced DEGs with genes differentially expressed between male (M) and female (F) animals in each group. DEGs from days 1 and 3 after vaccination were combined into a single list for this analysis. [file mmc7.pdf]
