# Supplementary material for: Preclinical development of a replication-competent vesicular stomatitis virus-based Lassa virus vaccine candidate advanced into human clinical trials
Source: eBioMedicine. 2025 Mar 28;114:105647. doi: 10.1016/j.ebiom.2025.105647 (PMC11994357; doi:10.1016/j.ebiom.2025.105647)
Supplement: Supplementary Table S1 [file mmc8.docx]

**Table S1.** VSV∆G-LASV-GPC RNAemia detected by RT-qPCR.

| **Vaccine** | **N=** | **Dose** | **C_t_ values on day after vaccination** | | |
| --- | --- | --- | --- | --- | --- |
|  |  |  | **d1** | **d3** | **d7** |
| **Control** | 3 | NA | ≥ 36 | ≥ 36 | ≥ 36 |
| **VSV∆G-LASV-GPC** | 5 | 2x10^7 | ≥ 36 | ≥ 36 | ≥ 36 |
| **VSV∆G-LASV-GPC** | 5 | 2x10^5 | ≥ 36 | ≥ 36 | ≥ 36 |

Samples with C_t_ values ≥ 36 after RT-qPCR are below the limit of detection. Day of vaccination is d0.
