# Supplementary material for: Preclinical development of a replication-competent vesicular stomatitis virus-based Lassa virus vaccine candidate advanced into human clinical trials
Source: eBioMedicine. 2025 Mar 28;114:105647. doi: 10.1016/j.ebiom.2025.105647 (PMC11994357; doi:10.1016/j.ebiom.2025.105647)
Supplement: Supplementary Table S2 [file mmc9.docx]

**Table S2:** Genbank accession numbers for LASV GPC and MARV GP sequences used as the source for developing VSV∆G chimeras used in the virus neutralization assay.

| **Virus** | **Strain** | **Origin** | **Genbank** |
| --- | --- | --- | --- |
| **LASV I** | Pinneo-NIG-1969 | Nigeria | KM822128 |
| **LASV II** | LASV237-NIG-2010 | Nigeria | AIT17638 |
| **LASV III** | Nig08-A19 | Nigeria | GU481072 |
| **LASV IV** | Josiah | Sierra Leone | HQ688672 |
| **LASV V** | Soromba-R | Mali | KF478765 |
| **LASV VI** | Not used | Nigeria (*Hylomyscus pamfi* isolate) |  |
| **LASV VII** | TGO/2016/812939 | Togo | MF990889 |
| **MARV** | Musoke | Kenya | ABA87127.1 |
